# Genetic polymorphisms of the *IL6* and *NOD2* genes are risk factors for inflammatory reactions in leprosy

**DOI:** 10.1371/journal.pntd.0005754

**Published:** 2017-07-17

**Authors:** Carolinne Sales-Marques, Cynthia Chester Cardoso, Lucia Elena Alvarado-Arnez, Ximena Illaramendi, Anna Maria Sales, Mariana de Andréa Hacker, Mayara Garcia de Mattos Barbosa, José Augusto da Costa Nery, Roberta Olmo Pinheiro, Euzenir Nunes Sarno, Antonio Guilherme Pacheco, Milton Ozório Moraes

**Affiliations:** 1 Laboratório de Hanseníase, Instituto Oswaldo Cruz, FIOCRUZ, Rio de Janeiro, RJ, Brazil; 2 Programa de Computação Científica (PROCC), FIOCRUZ, Rio de Janeiro, RJ, Brazil; Johns Hopkins Bloomberg School of Public Health, UNITED STATES

## Abstract

The pathways that trigger exacerbated immune reactions in leprosy could be determined by genetic variations. Here, in a prospective approach, both genetic and non-genetic variables influencing the amount of time before the development of reactional episodes were studied using Kaplan–Meier survival curves, and the genetic effect was estimated by the Cox proportional-hazards regression model. In a sample including 447 leprosy patients, we confirmed that gender (male), and high bacillary clinical forms are risk factors for leprosy reactions. From the 15 single nucleotide polymorphisms (SNPs) at the 8 candidate genes genotyped (*TNF*/*LTA*, *IFNG*, *IL10*, *TLR1*, *NOD2*, *SOD2*, and *IL6)* we observed statistically different survival curves for rs751271 at the *NOD2* and rs2069845 at the *IL6* genes (log-rank p-values = 0.002 and 0.023, respectively), suggesting an influence on the amount of time before developing leprosy reactions. Cox models showed associations between the SNPs rs751271 at *NOD2* and rs2069845 at *IL6* with leprosy reactions (HR_GT_ = 0.45, p = 0.002; HR_AG_ = 1.88, p = 0.0008, respectively). Finally, IL-6 and IFN-γ levels were confirmed as high, while IL-10 titers were low in the sera of reactional patients. Rs751271-GT genotype-bearing individuals correlated (p = 0.05) with lower levels of IL-6 in sera samples, corroborating the genetic results. Although the experimental size may be considered a limitation of the study, the findings confirm the association of classical variables such as sex and clinical forms with leprosy, demonstrating the consistency of the results. From the results, we conclude that SNPs at the *NOD2* and *IL6* genes are associated with leprosy reactions as an outcome. *NOD2* also has a clear functional pro-inflammatory link that is coherent with the exacerbated responses observed in these patients.

## Author summary

Leprosy reactions are abrupt inflammatory episodes that can occur before, during or after treatment. Although reactional episodes are the main cause of physical disabilities in patients, there are few genetic studies evaluating this outcome. We studied the influence of both risk factors and genetic markers in leprosy reaction outcomes. For this, we used a population including patients that either did or did not develop a reaction, and we performed a characterization of the genetic markers indicating susceptibility to leprosy. As a result, we identified how variables such as gender and clinical forms influence the amount of time before the occurrence of a reaction. In addition, we highlighted genetic markers related to the timing of the reaction’s occurrence, and associated them with reaction susceptibility. These markers were also related to the production of anti- and pro-inflammatory cytokines. Our study describes the risk factors and genetic components associated with leprosy reactions and can help in the prognosis and monitoring of patients to prevent clinical complications.

## Introduction

Leprosy reactions affect up to 50% of patients [1]. They are episodes that disrupt the natural course of disease, characterized by a strong and abrupt reactivation of immune responses [2–4]. Generally, nerve injury is present, which may lead to permanent incapacities. At the onset of the reactions, high levels of cytokines such as IL-1, IL-6, IL-12, IFN-γ, and TNF [2,5–12] have been detected either in serum or skin lesions, and recently, cytokine profiling has identified promising host biomarkers to reaction in patients from Bangladesh, Brazil, Ethiopia, and Nepal [13]. Also, anti-LID-1 antibody levels were found to be high and persistent in multibacillary leprosy patients who developed a reaction, therefore suggesting that this is a putative serological predictive tool [14].

Reactions can be observed prior to, during, or after multidrug therapy (MDT) and are classified as type 1, or reversal reaction (T1R), and type 2, or erythema nodosum leprosum (T2R). T1R is common among patients exhibiting *borderline* clinical forms of leprosy and consists of an increased cell-mediated immune response with intense skin and nerve inflammation [15,16]. T2R is mainly observed in patients at the lepromatous pole, and is related to acute cellular immune response activation, leading to severe systemic symptoms [17,18]. Epidemiological studies [19] suggest that comorbidities and pregnancy are risk factors for reaction outcome [20,21]. In addition, medical variables including clinical form, positive bacillary index, introduction of multidrug therapy, age, and gender also play key roles in leprosy reactions [22–24]. The genetic component influencing leprosy reactions, severe nerve impairment, and/or disabilities has been previously suggested [25]. Remarkable similarities were detected between granulomatous inflammatory responses and the presence of polymorphisms at genes such as *NOD2* and *LRRK2* associated with either leprosy reactions and other inflammatory diseases such as Crohn’s, ulcerative colitis (UC), and inflammatory bowel disease (IBD) [26,27]. SNPs at *NINJ1*, *TLR1*, *IL6*, and *TNFSF8* are clearly associated with reactional phenotypes (TR1 or TR2) [28–32]. Recently, the first GWAS assessing reaction outcome has identified *lncRNA* as a risk factor to T1R [33]. However, so far few of these genes have been replicated or functionally characterized to support the epidemiological findings.

The present study was designed to investigate by means of survival curves the risk factors associated with reactional episodes by testing Brazilian leprosy patients and the effects of 15 SNPs at 8 genes. The markers included 11 SNPs selected to replicate the previously reported associations of the *TLR1*, *NOD2*, and *IL6* genes and the remaining SNPs in 4 candidate genes *TNF*, *LTA*, *IFNG*, and *IL10*, in reference to markers previously associated with leprosy *per se* as an outcome [30,31,34–37]. Here, we confirmed the association between *IL6* and *NOD2* SNPs and leprosy reactions among Brazilians, and demonstrated that the *NOD2* genotype associated with a decreased risk for developing a reaction is also related to lower levels of IL-6 in the sera of non-reactional patients.

## Methods

### Ethics statement

Written informed consent was obtained from all individuals included in the study as required by the Research Ethics Committee at Fiocruz (CEP-Fiocruz Protocol 151/01). Our institutional ethical committee board allowed to disclose data as de-identified dataset information regarding clinical parameters and genetic/functional data specific to the research article.

### Subjects and study design

We performed a study including patients with a confirmed leprosy diagnosis between 1985 and 2008 that attended Souza Araújo Outpatient Reference Unit, Fiocruz, Rio de Janeiro, who were followed for the development of a leprosy reaction (outcome). Experienced professionals performed the leprosy and leprosy reaction diagnoses after careful clinical examinations and histopathological analyses. Leprosy patients were classified according to Ridley–Jopling criteria [38]: I, indeterminate; TT, tuberculoid; BT, borderline tuberculoid; BB, borderline borderline; BL, borderline lepromatous; LL, lepromatous leprosy, and were treated as specified by the World Health Organization (WHO) according to the multibacillary (MB) and paucibacillary (PB) classifications. Reaction occurrence, as well as classification as either T1R or T2R, were determined by clinical examination and confirmed by histopathological evaluation [15,39]. Follow-up started upon initiation of leprosy treatment (MDT/WHO) and stopped on the day of the first reaction episode (event) or the date of the last follow-up available. When the date of the last follow-up was not available, we limited the observation time to 3 years after start of follow-up. Therefore, we defined the patients from this group as not reactional, considering that the great majority of the patients who develop reaction do so in the first year after the diagnosis. We excluded patients classified as TT since they are not at risk of developing reactions and also excluded individuals without available information regarding their treatment dates. Additional variables such as gender, age, ethnicity, and leprosy relapse were retrieved from each patient’s medical record. For the cytokine quantification (functional study), we used serum from a group of 84 leprosy patients diagnosed at the same outpatient unit. All the characteristics of the first-time patients diagnosed with leprosy are detailed in the S1 Table.

### Selection of polymorphisms

The present study was designed to investigate the association of *TNF/LTA*, *IL10*, *IFNG*, *IL6*, *TLR1*, and *NOD2* genes with leprosy reactions. The SNPs *TNF* -308G>A (rs1800629), *LTA* +252A>G (rs909253), *IL10* -819C>T (rs1800871), and *IFNG* +874T>A (rs2430561) were selected based on previously published data which showed their association to leprosy *per se* among Brazilians [34,35,40]. The SNPs rs2069832, rs2069840, and rs2069845 were selected as tags that covered haplotype bins at *IL6* locus. The tag SNP search was based on data from the HapMap Genome Browser release #27 (http://hapmap.ncbi.nlm.nih.gov/) using Caucasians or African reference populations (CEU and YRI, respectively). The same strategy led to the selection of the SNPs rs751271, rs2066843, and rs748855 at the *NOD2* gene. The remaining *NOD2* SNPs (rs7194886, rs9302752, and rs8057341) were marker candidates retrieved from the literature [41]. *TLR1* SNPs rs5743592 and rs4833095 were included in this study in order to replicate previous findings regarding their association to leprosy *per se* and reactional episodes [29,30,42–44].

### DNA extraction and SNP genotyping

DNA was extracted from frozen blood samples using a salting-out precipitation method [45]. SNPs at *TNF/LTA*, *IFNG*, *IL10*, and *TLR1* loci were genotyped as described [34,35,40,42]. SNPs at the *NOD2* and *IL6* genes were genotyped using TaqMan^®^ assays according to the manufacturer’s instructions (Life Technologies, CA, USA). Briefly, amplifications were carried out in a final volume of 5 μL containing 2.5 μL of the TaqMan^®^ Universal Master Mix, 0.125 μL of the TaqMan mix (primers and probes), and 20–50 ng of template. PCR reactions were performed on ABI Prism 7000 and StepOne Plus Sequence Detection Systems (Life Technologies, CA, USA). Individuals with missing genotypes were excluded from the statistical analysis.

### Enzyme-linked immunosorbent assay (ELISA)

Serum cytokine levels from leprosy patients were quantified by ELISA, using Millipore’s MILLIPLEX® Human Cytokine/Chemokine panel commercial kit, according to the manufacturer’s instructions. The following cytokines were included in the kit: IL1-β, IL-4, IL-6, IL-10, IFN-γ, IL-12p40, IL-12P70, IL-13, IL-17, TNF-α.

### Statistical analysis

First, we performed a descriptive analysis to compare the influence of the considered variables on the length of time before the development of a leprosy reaction using survival curves. Proportional hazards assumption for different covariates in the Cox regression model was tested using Schoenfeld residuals [46]. All variables were adequate in the model, except for the clinical forms variable, which was added as strata in the model to avoid the effect of non-proportionality over time in the analyses. The genotype curves were obtained by the Kaplan–Meier method and compared using the log-rank statistic. Age at leprosy diagnosis (continuous variable categorized as ≤ 40 years old and > 40 years old), gender, ethnicity, leprosy relapse, and clinical forms were the analyzed variables. Leprosy clinical forms were grouped into three levels according to the risk of developing leprosy reactions: I/BT/BB, BL, and LL. The median time until event (MST) was calculated for each variable using Kaplan-Meir method. Then, to estimate the associations between genetic markers and leprosy reaction (outcome), crude and adjusted hazard ratios (HR and aHR, respectively) at 95% confidence interval and p-value were calculated using the Cox proportional-hazards regression model. Variables that showed significance with a reaction outcome in the survival curves were used to adjust the HR in the Cox model (possible confounders). For the cytokine measurements, the median values from each genotype group were compared by the Mann–Whitney U test (two groups of comparison) or by an ANOVA Kruskal–Wallis test (three groups). Missing data was excluded from the analysis. The statistical analyses for the Kaplan–Meier and Cox proportional-hazards regression model, as well as the survival graphical curves, were performed in the R environment for Windows [47], version 3.1.2, using the packages “survival” and “genetics.” The cytokine analysis was conducted using GraphPad Instat software (GraphPad Software 3.0 for Windows, San Diego, CA) and considered statistical significance to be a p-value < 0.05.

## Results

### Patients and clinical outcomes

Of 567 potentially eligible patients, 120 were excluded due to missing follow-up information, which are described in S2 Table. Therefore, a total of 447 patients were enrolled in the genetic study among whom 222 developed leprosy reactions with an overall median survival time (MST) of 165 weeks until the reaction’s occurrence (Table 1, S1A Fig). The clinical characteristics of the patients enrolled in the study, including their age, gender, ethnicity, leprosy relapse, and clinical classification are summarized in S1 Table and Table 1, along with the results of the log-rank test.

**Table 1:** Clinical characteristics of leprosy patients according to leprosy reaction outcome.

	All Patients* (N = 447)	No reaction* (N = 225)	Reaction* (N = 222)	MST (165 weeks)	Log-rank test (p-value^a^)
**Age at leprosy diagnosis**					0.429
≤ 40	230 (51.4)	110 (48.8)	120 (54.0)	148	
> 40	217 (48.5)	115 (51.2)	102 (46.0)	170	
**Gender**					0.002
Female	174 (38.9)	102 (45.3)	72 (32.4)	182	
Male	273 (61.1)	123 (54.7)	150 (67.6)	86	
Ethnicity**					0.987
Caucasoid	159 (53.7)	84 (53.2)	75 (54.3)	195	
Mestizo	111 (37.5)	60 (37.9)	51 (36.9)	182	
Black^b^	26 (8.8)	14 (8.9)	12 (8.8)	N/A	
Leprosy relapse**					0.822
No	442 (99.1)	223 (99.1)	219 (99.0)	165	
Yes	4 (0.9)	2 (0.09)	2 (1.0)	95	
**Clinical form group**					< 0.001
*I/BB/BT*	238 (53.2)	175 (77.7)	63 (28.4)	182	
*BL*	88 (19.7)	30 (13.3)	58 (26.1)	59	
*LL*	121 (27.1)	20 (9.0)	101 (45.5)	39	

* Results are shown as N (%).

** The number of subject counts in ethnicity and leprosy relapse may differ from the total individuals due to missing information. Abbreviations: MST, median survival time; I, indeterminate leprosy; BT, borderline tuberculoid; BB, borderline borderline; BL, borderline lepromatous; LL, leprosy lepromatous.

^a^ P- values were calculated using the log-rank test.

^b^ Censored category due to loss of follow-up.

As observed, the length of time before a leprosy reaction, as described by survival curves, shows a significant difference when considering the variables of gender (p = 0.002) and clinical form (p < 0.001). Regarding the median time until event (MST), men developed reactions earlier than women (MST = 86 weeks and 182 weeks, respectively). According to the clinical forms, the lower median was observed among LL patients when compared to the other groups (MST_LL_ = 39 weeks *vs* MST_BL_ = 59 weeks and MST_I/BB/BT_ = 182 weeks), as presented in Table 1, S1B and S1C Fig.

### Genetic association with leprosy reaction

To investigate the association between genetic markers and reaction outcomes we constructed survival curves for each SNP (stratified by genotype groups) as a descriptive approach to evaluate the occurrence of reaction in leprosy patients over time. We estimated that our sample was sufficient in detecting a genetic risk effect of 1.8 with a statistical power of 79%, considering single nucleotide variants with a minor allele frequency of 0.10 under the additive model.

**Fig 1:**
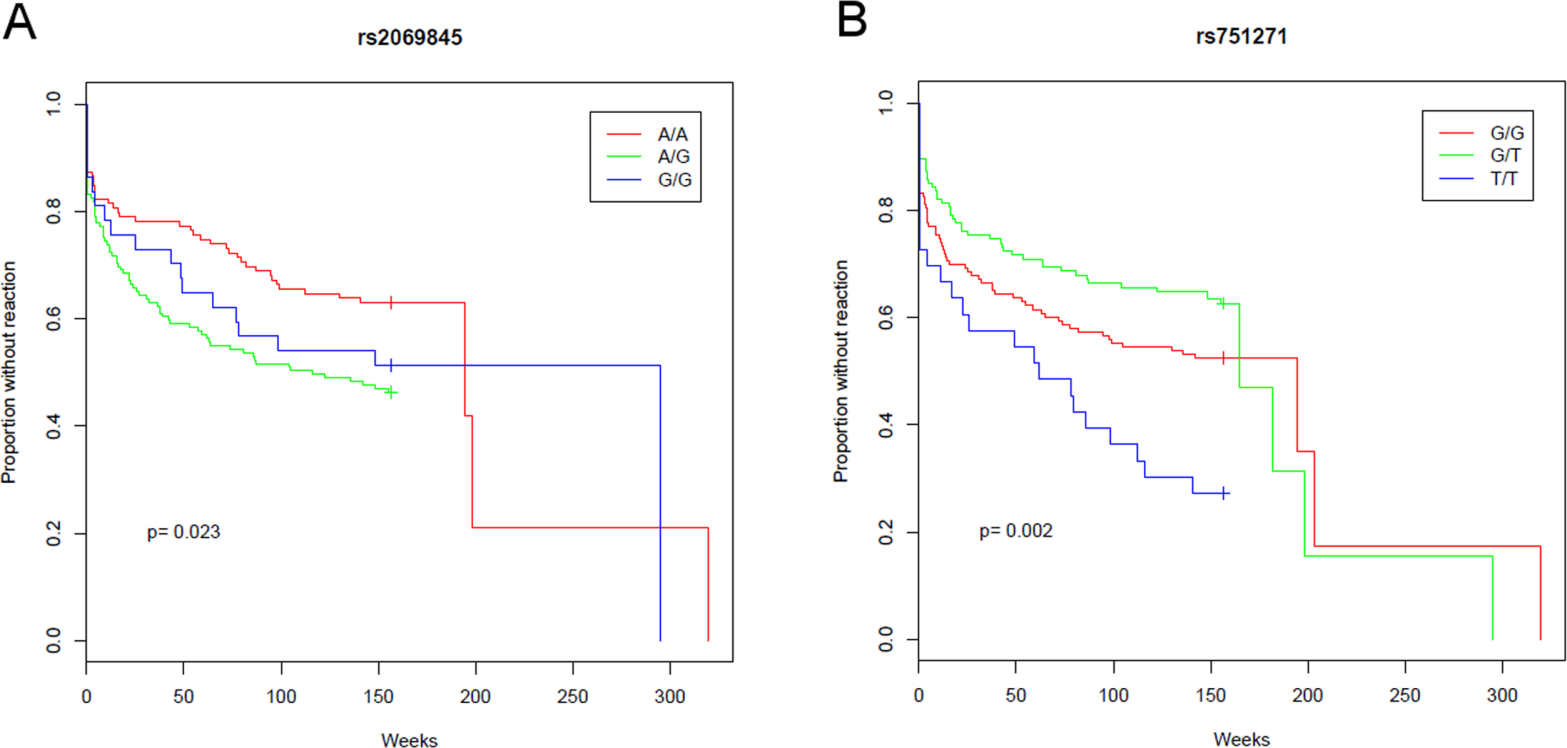
Survival curves of leprosy reactions by *NOD2* and *IL6* SNPs. Cumulative proportion of patients who developed leprosy reactions according to the *IL6* rs2069845 (A) and *NOD2* rs751271 (B) genotypes. The + symbol indicates censured samples. P-values were determined by log-rank tests.

From the 15 candidate SNPs (total counts and frequencies detailed at S3 Table), 2 showed different Kaplan–Meier curves between genotype groups (log-rank p-value < 0.05). Patients with the AG genotype for *IL6* rs2069845 developed reactions faster (MST = 116 weeks) than other genotypes (MST_AA_ = 195, MST_GG_ = 295 weeks; p = 0.023), as illustrated in Fig 1A. In addition, *NOD2* rs751271 homozygous patients (TT) developed reactions significantly earlier (MST = 62 weeks) than the GT (MST = 165 weeks) and GG (MST = 194 weeks) genotypes (Fig 1B). The log-rank results from the Kaplan–Meier comparisons of all SNPs tested are presented in S4 Table.

To estimate the SNP effects on reaction occurrence, the Cox proportional-hazards regression model was applied including adjusted analyses for gender and clinical forms (strata). The *NOD2* rs751271-GT genotype and G-allele carriers (GT/GG) were associated with protection against a reaction’s occurrence, even after adjustment (aHR = 0.45, p = 0.002 and aHR = 0.56, p = 0.01, respectively), as presented in Table 2. On the other hand, AG genotype and AG/GG carriers of *IL6* rs2069845 were associated with having an increased risk of developing a reaction (aHR = 1.88, p = 0.0008 and aHR = 1.73, p = 0.002, respectively). Genotypic information for individual patients is detailed in S5 Table.

**Table 2:** Results of Cox proportional-hazards model for *NOD2* rs751271 and *IL6* rs2069845 polymorphisms.

		Unadjusted		Adjusted ^a^	
SNP	Genotype	HR	p-value (95% CI)	aHR	p-value (95%CI)
*NOD2* rs751271					
	TT	Reference	-	Reference	-
	GT	0.41	0.0003 (0.25–0.67)	0.45	0.002 (0.27–0.75)
	GG	0.55	0.01 (0.34–0.88)	0.68	0.12 (0.43–1.10)
	GT/GG	0.48	0.001 (0.31–0.74)	0.56	0.01 (0.36–0.89)
*IL6* rs2069845					
	AA	Reference	-	Reference	-
	AG	1.65	0.006 (1.14–2.38)	1.88	0.0008 (1.30–2.73)
	GG	1.38	0.23 (0.80–2.36)	1.31	0.32 (0.76–2.25)
	AG/GG	1.59	0.009 (1.12–2.26)	1.73	0.002 (1.21–2.46)

Abbreviations: HR, hazard ratio; aHR, adjusted hazard ratio; CI, confidence interval.

^a^ HR values adjusted by gender and clinical form (added as strata in the model).

### Polymorphisms and the activation of an inflammatory response

**Fig 2:**
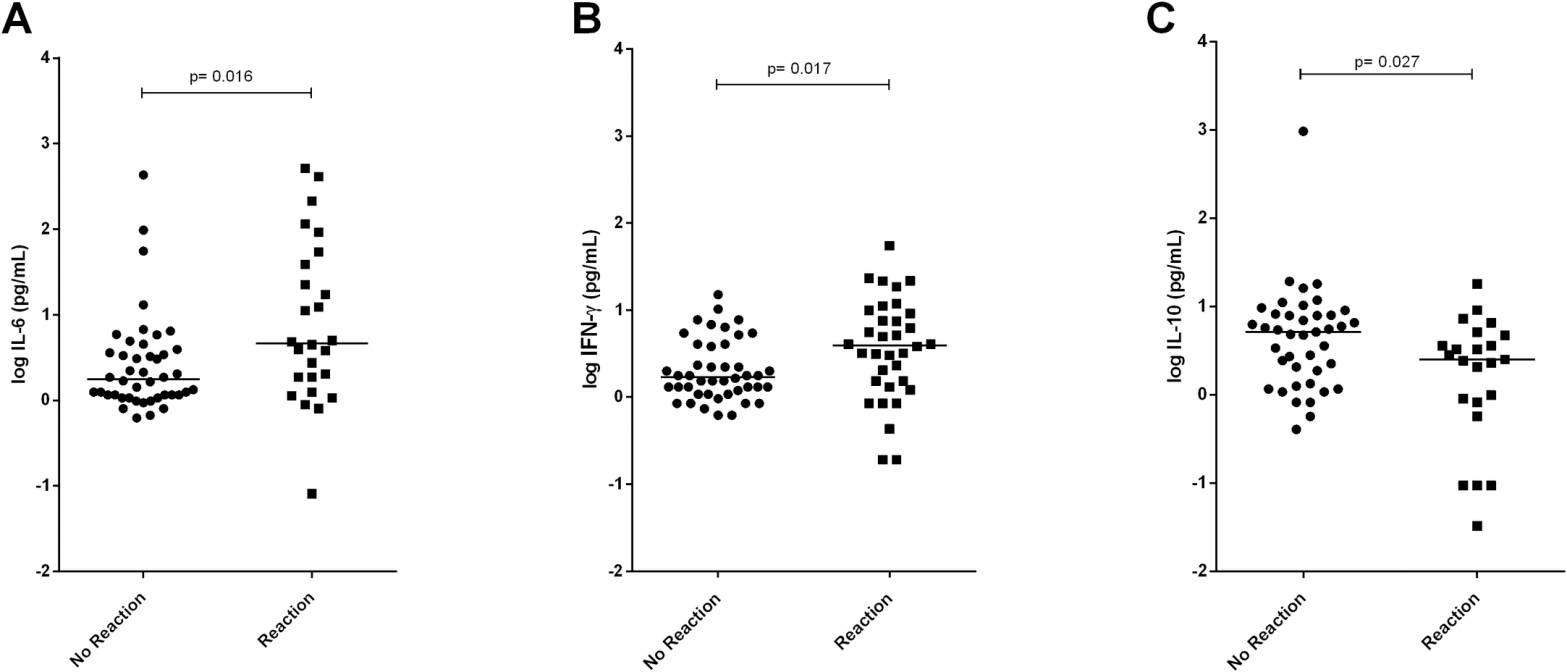
Serum dosage of IL-6, IFN-γ, and IL-10 and in leprosy patients that did not develop (No Reaction) or developed (Reaction) a reaction. (A) IL-6 dosage; No Reaction, N = 46; Reaction, N = 31. (B) IFN-γ dosage; No Reaction, N = 44; Reaction, N = 34. (C) IL-10 dosage; No Reaction, N = 41; Reaction, N = 23. The values were converted to a logarithmic scale. Cytokine production (pg/mL) was evaluated by ELISA and the median values were compared by a Mann–Whitney *t* test.

We evaluated serum cytokine levels to investigate whether the SNPs correlated with the inflammatory profile in leprosy patients. Of 84 individuals (S1 Table), 39 developed reactions, including 22 T1R and 17 T2R subtypes. Overall, IL-6, IL-10, and IFN-γ dosage (pg/mL) confirmed the literature reports in which reactional patients exhibited higher pro-inflammatory and lower anti-inflammatory cytokine levels (Fig 2). The quantification showed that IL-6 (median: reaction group 0.68 *vs*. no reaction group 0.25, p = 0.016) and IFN-γ (median: reaction group 0.61 *vs*. no reaction group 0.25, p = 0.017) were higher in the reaction group (Fig 2A and 2B). Also corroborating previous findings, IL-10 levels were lower in the reaction group (median: reaction group 0.40 *vs*. no reaction group: 0.71, p = 0.027), as shown in Fig 2C. The cytokines quantification for each of the patients analyzed in the functional approach are described in S6 Table. The remaining cytokines from the kit were not analyzed because they did not reach the detection limit.

**Fig 3:**
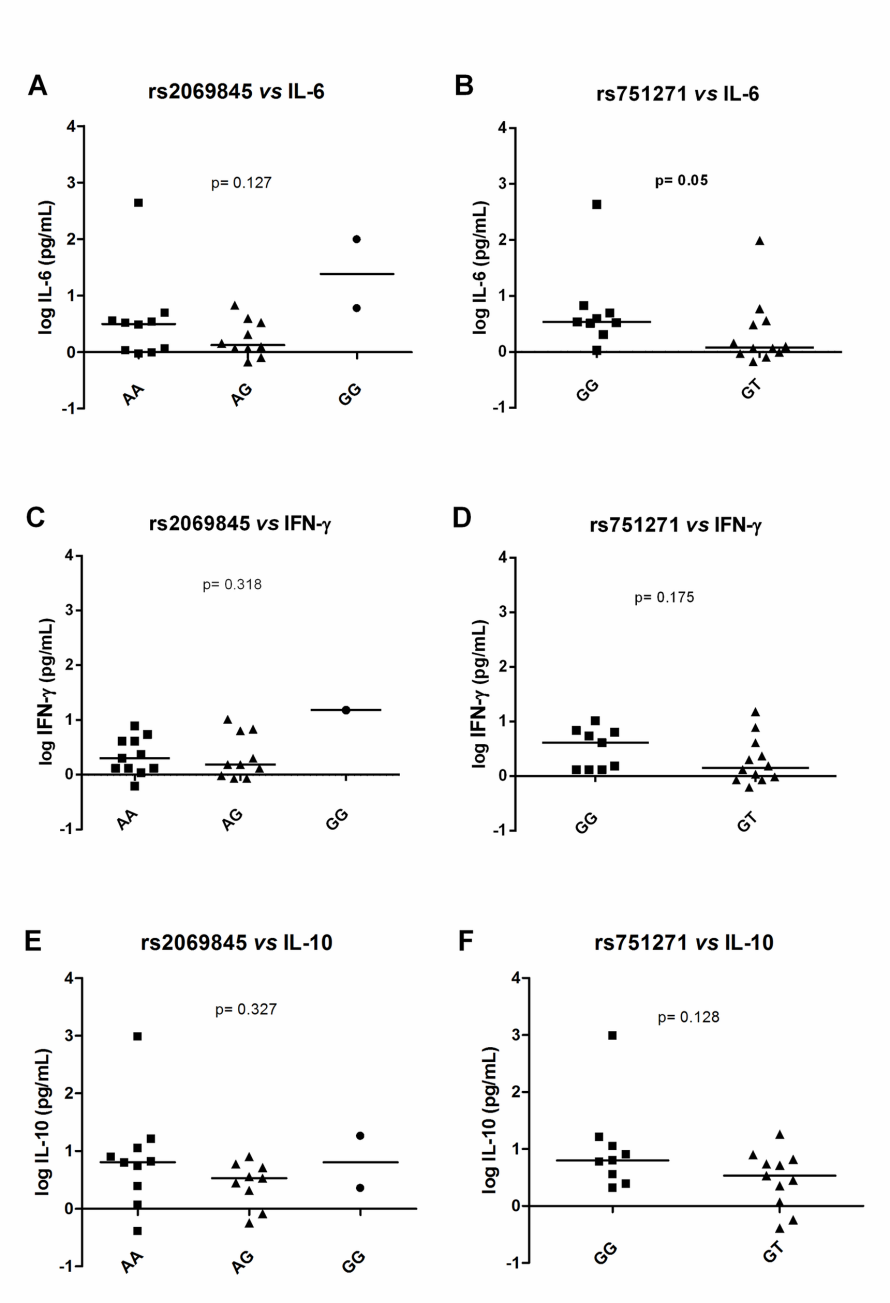
**IL-6 (A and B), IFN-γ (C and D) and IL-10 (E and F) dosage from non-reactional leprosy patients stratified by *IL6* (rs2069845) and *NOD2* (rs751271) polymorphisms**. Data are represented on a log scale. Cytokine production was evaluated by ELISA and the median values were compared by a Mann–Whitney *t* test. rs2069845 *vs*. IL-6: AA, N = 10; AG, N = 10; GG, N = 1. rs751271 *vs*. IL-6: GG, N = 9; GT, N = 12. rs2069845 *vs*. IFN-γ: AA, N = 11; AG, N = 9; GG, N = 2. rs751271 *vs*. IFN-γ: GG, N = 9; GT, N = 12. rs2069845 *vs*. IL-10: AA, N = 10; AG, N = 9; GG, N = 2. rs751271 *vs*. IL-10: GG, N = 9; GT, N = 11.

Then, the cytokine levels from patients were stratified in accordance with *IL6* and *NOD2* SNPs (Fig 3). The cytokine dosage showed a heterogeneous distribution among the patients, and stratified analysis showed no differences between groups in either the patients overall (S2 Fig) or the reactional patients (S3 Fig). Considering the non-reactional leprosy patients, IFN-γ and IL-10 levels showed no differences between genotype groups for either of the tested polymorphisms (Fig 3C–3F). Neither was IL-6 production associated with *IL6* rs2069845 SNP (Fig 3A). However, individuals carrying *NOD2* rs751271-GT showed a borderline difference (p = 0.05), suggesting lower IL-6 production (GT median: 0.008) as compared to GG individuals (GG median: 0.53) (Fig 3B). Unfortunately, we did not have patients with the *NOD2* rs751271-TT genotype for a functional analysis.

## Discussion

In the present study, we have found two polymorphisms that, independently of other non-genetic risk factors, were associated with inflammatory reactions in leprosy. Considering that reactions are serious, and remain an unpredictable outcome, we decided to include the time of occurrence in the model using survival analysis, which tests the prognostic value of polymorphisms as genetic markers. As a result, we observed that patients with *NOD2* rs751271-TT or carrying an *IL6* rs2069845-G allele developed reactions faster than those with other genotypes/alleles, suggesting that these SNPs are good prognostic markers for reactional episodes. Moreover, *NOD2* rs751271-GT individuals had a statistically significant decreased risk of developing reactions and, in a smaller group of patients, also showed lower serum IL-6, but not IL-10 or IFN-γ, levels.

**Fig 4:**
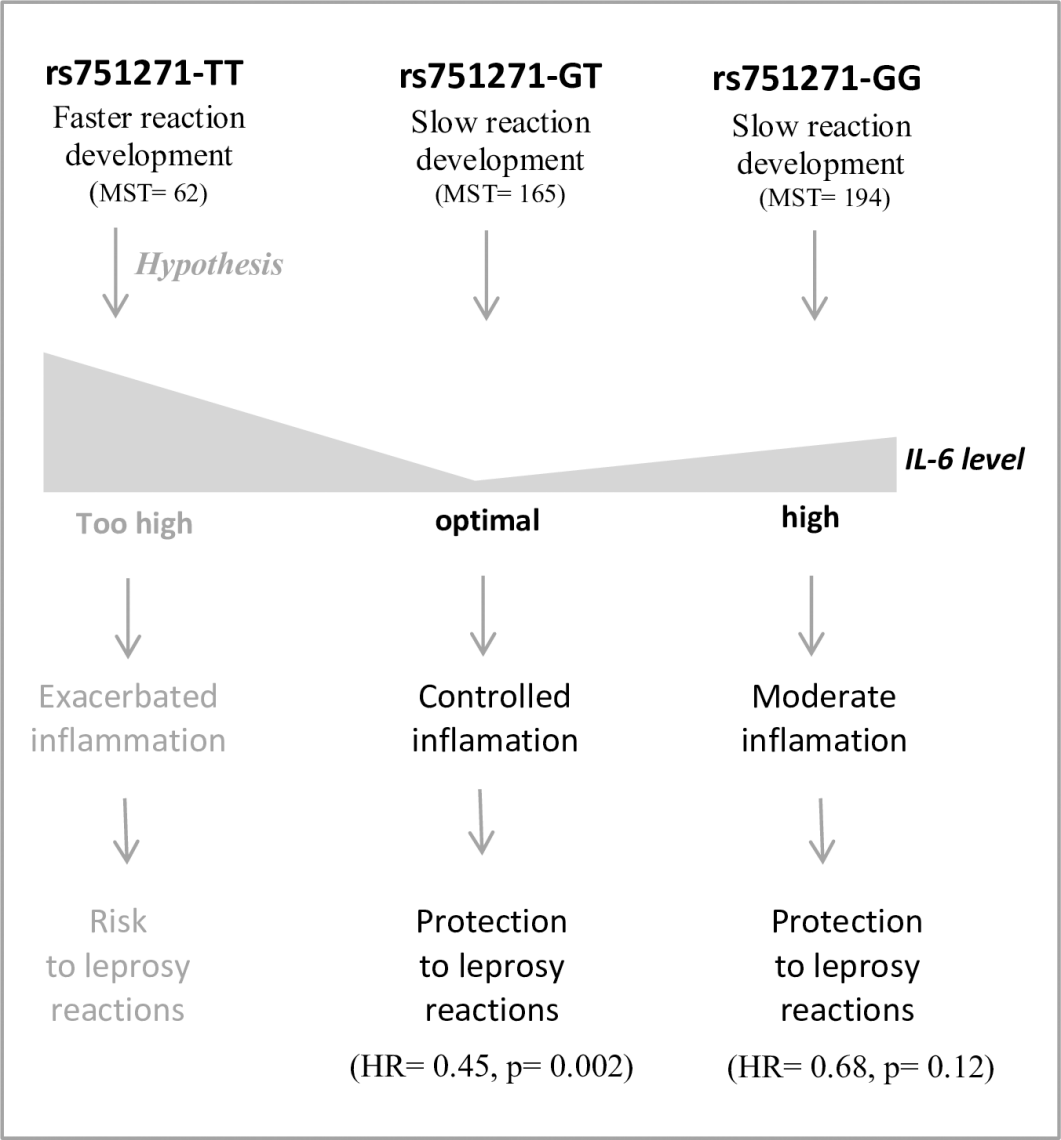
Graphical abstract to illustrate a possible effect of *NOD2* rs751271 SNP in leprosy reactions. Patients with the rs751271-GT genotype had lower IL-6 levels, which could influence the inflammatory balance and the susceptibility to or protection against leprosy reactions. NS: Not statistically significant.

SNPs at *NOD2* were previously associated with protection against reaction outcomes in a population from Nepal [36]. NOD2 acts as a sensor of mycobacterial components and contributes to bacterial killing by activating the NF-kB pathway, an inflammation cascade, and an interleukin-32-dependent pathway. Also, NOD2 appears as a key player in autophagy, including its genetic association with Crohn’s disease [48–50]. Uncontrolled inflammation is driven by an inability to clear bacteria through autophagy in patients carrying specific alleles, and that is followed by exacerbated pro-inflammatory response. In fact, here, *NOD2* rs751271-TT individuals have a higher risk of developing a reaction *per se*. None of the individuals enrolled in the functional analysis exhibited the TT genotype. Our results suggest that rs751271-GT individuals have the lowest risk of a premature reactional outcome and these individuals have lower productions of IL-6 during an unreactional state. It could be hypothesized that rs751271-GT carriers have the most balanced production of cytokines—in this case IL-6 that could be used as a readout—and during the natural course of leprosy (in non-reactional patients), these patients showed a decreased risk of experiencing a reactional episode. On the other hand, rs751271-GG individuals have higher IL-6 and a moderate risk of developing reactions (survival curves). TT individuals would possibly exhibit a similar high IL-6 production pro-inflammatory profile, although unfortunately we did not have any patients with this genotype in our sample so we could not perform such a comparison. This hypothesis is illustrated in Fig 4.

The results from different studies have demonstrated the emergence of an inflammatory immune response during reactional episodes [4], and a pro-inflammatory signature to reactions was suggested recently by Khadge and colleagues [13]. Probably, polymorphisms are contributing to control the inflammatory profile in leprosy patients, influencing the reaction’s development, when it occurs, and its severity.

*IL6* has been previously associated with leprosy reactions in other populations and by using different study designs. The *IL6* rs2069840-G allele was associated with protection against and *IL6* rs2069845-G with risk of T2R in a population from Goiania, Brazil [31], which is, at least in part, corroborated by our genetic results indicating that *IL6* rs2069845-G is associated with an increased risk of reactional episodes *per se*. IL-6 is a pleiotropic cytokine that acts in the acute inflammatory response and activation of Th1 and Th17 lymphocytes. IL-6 also inhibits T regulatory (Treg) cells, mediating the balance between pro-inflammatory and immunosuppressive T-cells [51], which is consistent with the central role of IL-6 as a prognostic molecule in chronic inflammation. Our data confirmed higher levels of IL-6 among reactional patients, but a stratification of IL-6 genotypes failed to confirm that IL-6 rs2069845-G carriers secrete more IL-6 in serum. In fact, SNPs only partly and transiently regulate gene expression and maybe the period for the detection of IL-6 in the sera that could be impacted by SNPs may be different from what we recovered in the present study.

We have additionally investigated the classic candidate genes *TNF/LTA*, *IFNG*, and *IL10*, which were consistently associated with leprosy *per se* [35,40,52] but, despite the central roles of these cytokines in leprosy reactions [5], were not genetically associated with reaction outcomes in our study. We were also unable to detect any association of *TLR1* SNPs, although the previously reported data regarding the association of this gene in other populations [30]. Variations in study designs and linkage disequilibrium patterns may explain these divergent results.

Recently, SNPs at *TNFSF* pathways were consistently associated with T1R outcomes in populations from Vietnam and Brazil [32,53]. These and other possible candidates still need to be confirmed among our Southern Brazilian population. Also, it is necessary to understand the mechanisms that underlie the genetic susceptibility of reactional episodes since in this study we observed that the presence of risk alleles do not have additive or synergistic effects in a way that would make the combination of some of these most important SNPs as a genetic test feasible. We understand that the sample size could be considered a limitation of our study, mainly due to low patient compliance, however, the results confirm certain classical variables (gender and clinical forms) as risk factors for reaction, which implies that our genetic data is consistent and should be used in a score to estimate the risk of multibacillary patients developing reactional episodes. Also, we have to consider other possible sources of bias such as non-genetic variables that could determine the development of reactions for which we did not have any available information, amongst them pregnancy, modifications in therapeutic scheme, and comorbidities. Nevertheless, the specific variables for which we have information were utilized as covariates and adjusted for in the Cox model.

The issue of correcting for multiple comparisons has been recently discussed in epidemiology. Even though it is desirable to control for type I error, there are many instances when it is not sound to consider that a priori all the hypotheses being tested are false. As it is our case we have performed a genetic study at candidate gene/SNPs level, we did not consider it applicable to perform corrections for multiple comparisons, since it could increase the Type II error and not finding a true association [54].

Despite the studies suggesting the influence of host and environmental factors on leprosy reactions, the complete mechanisms of their occurrence remain unclear. However, there is no doubt that reactions are the main cause of physical disabilities. Results obtained from a prospective study on leprosy patients from Brazil showed that 30% of the reactional cases were associated with persistent physical impairment [55]. The development of a prognostic panel with the capacity to predict the likelihood of progressing to reactions indicates a possible strategy that could contribute to the surveillance of patients with greater chances of developing clinical complications and possibly interfere with prophylaxis in order to prevent future disabilities.

## Supporting information

S1 Checklist STROBE checklist for cohort study including information about the present study. (PDF)

S1 Fig Non-genetic variables affecting reaction outcome. Cumulative proportion of reaction occurrence among leprosy patients overall (A), leprosy patients by gender (B), and by clinical forms (C). Kaplan–Meier curves were compared using a log-rank test. The + symbol indicates censured samples. P = p-value from a log-rank test. F = female, M = male, I = indeterminate leprosy, BT = borderline tuberculoid, BB = borderline borderline, BL = borderline lepromatous, LL = leprosy lepromatous.
(TIF)

S2 Fig Cytokine levels in leprosy patients: Non-carriers and carriers of the minor allele of *IL6* rs2069845 and *NOD2* rs751271 SNPs. IL-6 (A and B), IFN-γ (C and D), and IL-10 (E and F) levels were quantified in the serum of leprosy patients using enzyme-linked immunosorbent assay (ELISA). Median dosage values were compared between groups by a Mann–Whitney *t* test. Sample size (N): A) AA = 18, AG+GG = 18; B) GG = 14, GT+TT = 21; C) AA = 22, AG+GG = 21; D) GG = 17, GT+TT = 21; E) AA = 17, AG+GG = 20; F) GG = 15; GT+TT = 20.
(TIF)

S3 Fig Cytokine levels in reactional leprosy patients: Non-carriers and carriers of the minor allele of *IL6* rs2069845 and *NOD2* rs751271 SNPs. IL-6 (A and B), IFN-γ (C and D), and IL-10 (E and F) levels were quantified in the serum from reactional leprosy patients using enzyme-linked immunosorbent assay (ELISA). Median dosage values were compared between groups by a Mann–Whitney *t* test. Sample size (N): (A) AA = 8, AG+GG = 8; (B) GG = 5, GT+TT = 9; (C) AA = 11, AG+GG = 11; (D) GG = 9, GT+TT = 13; (E) AA = 7, AG+GG = 9; (F) GG = 6, GT+TT = 8.
(TIF)

S1 Table Demographic and clinical characteristics of patients in the first diagnosis of leprosy. (TIF)

S2 Table Description of excluded patients from survival analysis of leprosy genetic study. (TIF)

S3 Table Total count and frequencies for genotypic and minor allele carriers for each of the studied polymorphisms. (PDF)

S4 Table Associations between SNPs and time to leprosy reaction development. (TIF)

S5 Table Genotypic information for individual patients from the genetic study. (PDF)

S6 Table Cytokines quantification for each of the patients analyzed in the functional study. (PDF)
