# Supplementary material for: Genetic polymorphisms of the IL6 and NOD2 genes are risk factors for inflammatory reactions in leprosy
Source: PLoS Negl Trop Dis. 2017 Jul 17;11(7):e0005754. doi: 10.1371/journal.pntd.0005754 (PMC5531687; doi:10.1371/journal.pntd.0005754)
Supplement: S1 Checklist — (PDF) [file pntd.0005754.s001.pdf]

STROBE Statement—checklist of items that should be included in reports of observational studies

	Item No	Recommendation
**Title and abstract**	1	(*a*) Indicate the study's design with a commonly used term in the title or the abstract
		(*b*) Provide in the abstract an informative and balanced summary of what was done and what was found
**Introduction**		
Background/rationale	2	Explain the scientific background and rationale for the investigation being reported
Objectives	3	State specific objectives, including any prespecified hypotheses
**Methods**		
Study design	4	Present key elements of study design early in the paper
Setting	5	Describe the setting, locations, and relevant dates, including periods of recruitment, exposure, follow-up, and data collection
Participants	6	(*a*) *Cohort study*—Give the eligibility criteria, and the sources and methods of selection of participants. Describe methods of follow-up<br>*Case-control study*—Give the eligibility criteria, and the sources and methods of case ascertainment and control selection. Give the rationale for the choice of cases and controls<br>*Cross-sectional study*—Give the eligibility criteria, and the sources and methods of selection of participants
		(*b*) *Cohort study*—For matched studies, give matching criteria and number of exposed and unexposed<br>*Case-control study*—For matched studies, give matching criteria and the number of controls per case
Variables	7	Clearly define all outcomes, exposures, predictors, potential confounders, and effect modifiers. Give diagnostic criteria, if applicable
Data sources/ measurement	8*	For each variable of interest, give sources of data and details of methods of assessment (measurement). Describe comparability of assessment methods if there is more than one group
Bias	9	Describe any efforts to address potential sources of bias
Study size	10	Explain how the study size was arrived at
Quantitative variables	11	Explain how quantitative variables were handled in the analyses. If applicable, describe which groupings were chosen and why
Statistical methods	12	(*a*) Describe all statistical methods, including those used to control for confounding
		(*b*) Describe any methods used to examine subgroups and interactions
		(*c*) Explain how missing data were addressed
		(*d*) *Cohort study*—If applicable, explain how loss to follow-up was addressed<br>*Case-control study*—If applicable, explain how matching of cases and controls was addressed<br>*Cross-sectional study*—If applicable, describe analytical methods taking account of sampling strategy
		(*e*) Describe any sensitivity analyses

Continued on next page

**Results**		
Participants	13*	(a) Report numbers of individuals at each stage of study—eg numbers potentially eligible, examined for eligibility, confirmed eligible, included in the study, completing follow-up, and analysed
		(b) Give reasons for non-participation at each stage
		(c) Consider use of a flow diagram
Descriptive data	14*	(a) Give characteristics of study participants (eg demographic, clinical, social) and information on exposures and potential confounders
		(b) Indicate number of participants with missing data for each variable of interest
		(c) *Cohort study*—Summarise follow-up time (eg, average and total amount)
Outcome data	15*	*Cohort study*—Report numbers of outcome events or summary measures over time
		Case-control study—Report numbers in each exposure category, or summary measures of exposure
		Cross-sectional study—Report numbers of outcome events or summary measures
Main results	16	(*a*) Give unadjusted estimates and, if applicable, confounder-adjusted estimates and their precision (eg, 95% confidence interval). Make clear which confounders were adjusted for and why they were included
		(*b*) Report category boundaries when continuous variables were categorized
		(*c*) If relevant, consider translating estimates of relative risk into absolute risk for a meaningful time period
Other analyses	17	Report other analyses done—eg analyses of subgroups and interactions, and sensitivity analyses
**Discussion**		
Key results	18	Summarise key results with reference to study objectives
Limitations	19	Discuss limitations of the study, taking into account sources of potential bias or imprecision. Discuss both direction and magnitude of any potential bias
Interpretation	20	Give a cautious overall interpretation of results considering objectives, limitations, multiplicity of analyses, results from similar studies, and other relevant evidence
Generalisability	21	Discuss the generalisability (external validity) of the study results
**Other information**		
Funding	22	Give the source of funding and the role of the funders for the present study and, if applicable, for the original study on which the present article is based

*Give information separately for cases and controls in case-control studies and, if applicable, for exposed and unexposed groups in cohort and cross-sectional studies.

Note: An Explanation and Elaboration article discusses each checklist item and gives methodological background and published examples of transparent reporting. The STROBE checklist is best used in conjunction with this article (freely available on the Web sites of PLoS Medicine at http://www.plosmedicine.org/, Annals of Internal Medicine at http://www.annals.org/, and Epidemiology at http://www.epidem.com/). Information on the STROBE Initiative is available at www.strobe-statement.org.

ANSWERS TO STROBE CHECKLIST OF OBSERVATIONAL STUDY (COHORT STUDY)

STUDY: GENETIC POLYMORPHISMS OF THE *IL6* AND *NOD2* GENES ARE RISK FACTORS FOR INFLAMMATORY REACTIONS IN LEPROSY

1.

a. In the Title: GENETIC POLYMORPHISMS AT IL6 AND NOD2 GENES ARE RISK FACTORS FOR INFLAMMATORY REACTIONS IN LEPROSY. In the abstract: Line 33: “Here, in a prospective approach, the genetic and non-genetic influence in time until the development of reactional episodes were studied through Kaplan-Meier survival curves”

b. what was done: Lines 33-35: “Here, in a prospective approach, the genetic and non-genetic influence in time until the development of reactional episodes were studied through Kaplan-Meier survival curves, and genetic effect was estimated by Cox proportional hazards regression model”. Lines 37-39: “Then, 15 SNPs at seven candidate genes (*TNF*/*LTA*, *IFNG, IL10*, *TLR1, NOD2, SOD2* and *IL6)* were genotyped adjusting for gender and clinical forms”

- what was found: Lines 35-37: “In a sample including 447 leprosy patients, we confirmed that gender (male), and high bacillary clinical forms are related with leprosy reactions. “Lines 39-51: “We observed statistically different survival curves for rs721271 at NOD2 and rs2069845 at IL6 genes (log-rank p-value = 0.002 and 0.023, respectively), suggesting an influence in time until developing leprosy reactions irrespective of its nature - either reversal reaction (type I) or erythema nodosum leprosum (type II). Cox models showed that GT genotype and G allele of rs751271 at NOD2 were associated with protection against reaction (adjusted HR= 0.48, p= 0.003; adjusted HR= 0.58, p= 0.02). Conversely, patients carrying the AG genotype and the G allele of rs2069845 at IL6 showed increased risk of developing reactional episodes

(adjusted HR= 1.85, p= 0.001, adjusted HR= 1.69, p= 0.008). Finally, IL-6 levels were confirmed as higher, while IL-10 titers were low in the sera of reactional patients. rs751271 GT genotype-bearing individuals was correlated (p= 0.05) with lower levels of the log(IL-6) ratio in sera samples from unreactional patients that corroborates genetic results."

2. **Background:** Lines 82-90: " Leprosy reactions are episodes that disrupt the natural course of disease affecting 30-50% of the patients [1] and characterized by a strong and abrupt reactivation of the immune responses [2-4].(…) High levels of cytokines such as IL-1, IL-6, IL-12, IFN-γ and TNF [2,5-12] have been detected at the onset of the reactions either in serum or skin lesions, and recently cytokines profile were identified as promising host biomarkers to reaction in patients from Bangladesh, Brazil, Ethiopia and Nepal [13] (…) . Lines 91-98: Reactions can be observed prior, during or post multidrug therapy (MDT), and are classified in type 1 or reversal reaction (T1R), and type 2 or erythema nodosum leprosum (T2R). T1R is common among patients exhibiting *borderline* clinical forms (…) T2R is mainly observed among patients of the lepromatous pole, and is related to acute cellular immune response activation (…).

   Rational: Lines 97-109: Epidemiological studies [19] suggests that comorbidities and pregnancy are risk factors for reaction outcome [20-22]. (…) The genetic component influencing leprosy reactions, severe nerve impairment and/or disabilities has been suggested previously [25]. Remarkable similarities were detected between granulomatous inflammatory responses and the presence of polymorphisms at genes such as *NOD2* and *LRRK2* associated with either leprosy reactions and other inflammatory diseases (…) Other genes have been tested mainly in leprosy and have been associated with reactional phenotypes (TR1 or TR2) while SNPs at *NINJ1, TLR1, IL6,* and *TNFSF15* are clearly associated with reactions but results so far still lack replication studies or at least functional characterization to support the epidemiological findings.

3. Lines 110-115: The present study was designed to investigate by means of survival curves risk factors associated with reactional precipitation, while the effect of 15 SNPs at eight genes in leprosy reactions outcome testing Brazilian leprosy patients. The markers included 11 SNPs selected to replicate the previously reported associations of *TLR1*, *NOD2* and *IL6* genes and remaining SNPs in 4 candidates genes *TNF*, *LTA*, *IFNG* and *IL10*, previously associated with leprosy *per se* outcome

4. Lines 33-36: “Here, in a prospective approach, the genetic and non-genetic influence in time until the development of reactional episodes were studied through Kaplan-Meier survival curves, and genetic effect was estimated by Cox proportional hazards regression model”.

5. Lines 121-124: We performed a study considering as inclusion criteria patients with confirmed leprosy diagnosis between 1985 and 2008 that attended Souza Araújo Outpatient Reference Unit, Fiocruz, Rio de Janeiro, and that were followed for leprosy reaction development (outcome). Lines 132-134: “Follow up started at the date of leprosy treatment (MDT/WHO) initiation and stopped on the date of the first reaction episode (event) or the date of last available follow-up. Patients’ follow-up times were censored after 3 years.”

6.

- **a.** Lines 121-124: We performed a study considering as inclusion criteria patients with confirmed leprosy diagnosis between 1985 and 2008 that attended Souza Araújo Outpatient Reference Unit, Fiocruz, Rio de Janeiro, and that were followed for leprosy reaction development (outcome). Lines 131-137: “Follow up started at the date of leprosy treatment (MDT/WHO) initiation and stopped on the date of the first reaction episode (event) or the date of last available follow-up. Patients’ follow-up times were censored after 3 years. We excluded patients classified as TT since they are not at risk of developing reactions and also individuals without available date information for treatment. Additional variables such as gender, age, ethnicity and leprosy relapse were retrieved from each patient medical record.”

7. Outcome: lines 178-179: “Time until the development of reaction episodes was analyzed as outcome using survival methods”. Lines 183-186: “Age at leprosy diagnosis, gender, ethnicity, leprosy relapse and clinical form were also tested by the log-rank test. Variables that have shown significance with reaction outcome in the survival curves were used in the Cox model as covariates (possible confounders)”.

8. Lines 124-131: “Experienced professionals performed leprosy and leprosy reaction diagnosis after careful clinical examination. Leprosy patients were classified according to Ridley and Jopling criteria [28] - I, Intermediate; TT, Tuberculoid; BT, Borderline Tuberculoid; BB, Borderline Borderline; BL, Borderline Lepromatous; LL Lepromatous - and were treated as specified by the World Health Organization (WHO) according to multibacillary (MB) and paucibacillary (PB) classification. Reaction occurrence as well as the classification as T1R or T2R were determined by clinical examination and confirmed by histopathological evaluation”. Lines 136-137: “Additional variables such as gender, age, ethnicity and leprosy relapse were retrieved from each patient medical record”.

9. Lines 184-186: “Variables that have shown significance with reaction outcome in the survival curves were used in the Cox model as covariates (possible confounders).”

Line 381: “Also, we have to consider other possible sources of bias such as non-genetic variables that could determine the development of reaction for which we did not have available information, amongst them pregnancy, modifications in therapeutic scheme and comorbidities. Nevertheless the specific variables that we have information were utilized as covariates and adjusted for in the Cox analyses model."

10. Lines 197-199: “Of 567 potentially eligible patients, 120 were excluded due to missing follow-up information. Therefore a total of 447 patients were enrolled in the genetic study from which 222 developed leprosy reactions.”

11. Lines 184-186: For cytokine measurements, the median values from each genotype group were compared by Mann-Whitney U test.

12. .

- **a.** Lines 178-186: "Time until the development of reaction episodes was analyzed as outcome using survival methods. First, survival curves from genotypes, carriers of minor/major allele were obtained by Kaplan-Meier method, and compared using the log-rank test [40]. Crude and adjusted hazard ratios (HR) were calculated through Cox proportional hazard models and used to estimate associations between genetic markers and leprosy reaction (outcome). Age at leprosy diagnosis, gender, ethnicity, leprosy relapse and clinical form were also tested by the log-rank test. Variables that have shown significance with reaction outcome in the survival curves were used in the Cox model as covariates (possible confounders)."
- **b.** We did not examine interactions (please, see the answer 17).
- **c.** Line 190: "Missing data were excluded from the analysis."
- **d.** Lines 133-134: "The patients were censored after 3 years of follow-up".
- **e.** We did not perform sensitivity analyses (please, see the answer 17)

13.

a. and b. Lines 197-199: "Of 567 potentially eligible patients, 120 were excluded due to missing follow-up information. Therefore a total of 447 patients were enrolled in the genetic study from which 222 developed leprosy reactions…"

14. Table 1 (page 9), S1 Table (Supporting information) and Results section, lines 193-199 : "Of 567 potentially eligible patients, 120 were excluded due to missing follow-up information. Therefore, a total of 447 patients were enrolled in the genetic study among whom 222 developed leprosy reactions with an overall median survival time (MST) of 165 weeks until the reaction′s occurrence (Table 1, S1A Fig). The clinical characteristics of the patients enrolled in the study, including their age, gender, ethnicity, leprosy relapse, and clinical classification are summarized in S1 Table and Table 1, along with the results of the log-rank test".

15. Table 1, page 9 S1 Table (Supporting information).

16.

- **a.** Lines 202-205: "As observed, time until leprosy reaction, described by survival curves, shows significant differences between gender (p= 0.002) and clinical forms group (p<0.001). Therefore, these co-variates were selected to be included in the multivariate Cox model (Table 1)." See table 2, page 11.
- **b.** The variable "age of leprosy diagnosis" was the continuous variable that we used in the present work. We categorized it into ≤ 40 years age and > 40 years age. The category boundaries were described in the methodology, line 179: "Age at leprosy diagnosis (continuous variable categorized as ≤ 40 years old and > 40 years old) , gender, ethnicity, leprosy relapse and clinical forms were the analyzed variables"
- **c.** We used the HR (hazard ratio) as the estimate of risk, since it is the most adequate for the Cox model (survival analysis).

17. We are applying the survival model to genetic studies with polymorphisms initially as a descriptive model to evaluate the genetic influence on time of outcome occurrence (leprosy reaction). We expect to further extend the number of patients in order to perform additional statistical analyses including subgroups, interactions and sensitivity analyses.

18. Lines 283-293: "In the present study, we have found two polymorphisms that, independently of other non-genetic risk factors, were associated with inflammatory reactions in leprosy (...) As a result, we observed that patients with NOD2 rs751271-TT or carrying IL6 rs2069845-G allele developed reaction in a shorter time lapse compared to other genotypes/alleles, suggesting these SNPs as good prognostic markers for reactional episodes (...)".

19. Lines 377-385: "We understand that the sample size could be considered a limitation of our study, mainly due to low compliance of patients, although results are confirming classical variables (gender and clinical forms) as risk factors for reaction, which reinforces that our

genetic data is consistent and should be used in a score to estimate risk for multibacillary patients to develop reactional episodes. Also, we have to consider other possible sources of bias such as non-genetic variables that could determine the development of reaction for which we did not have available information, amongst them pregnancy, modifications in therapeutic scheme and comorbidities. Nevertheless the specific variables that we have information were utilized as covariates and adjusted for in the Cox analyses model."

20. Lines 302-305: Our results suggest that rs751271-GT individuals have the lowest risk towards prematurity of reactional outcome and these individuals have lower production of IL-6 in patients during unreactional state; lines 325-329: IL6 have been previously associated to leprosy reactions in other populations and using different study designs (...)

21. Lines 364-372: "Despite the studies suggesting the influence of host and environmental factors in leprosy reactions, the complete mechanisms of their occurrence remain unclear. However, there is no doubt that reactions are the main cause of physical impairment. Results obtained from a prospective study including leprosy patients from Brazil, showed that 30% of the reactional cases were associated with persistent physical impairment [54]. The development of a prognostic panel with predicting capacity of progressing to reactions indicates a possible strategy that could contribute in the surveillance of patients with higher chance to develop clinical complications and maybe to interfere with prophylaxis in order to prevent future disabilities".

22. We have included funding information in the paper: CSM was a Pos Doc funded by *Conselho Nacional de Desenvolvimento Científico (CNPq). MOM* is a *CNPq* and *Fundação de Amparo a Pesquisa do Estado do Rio de Janeiro (FAPERJ)* fellow. This study was supported by a grant from the Brazilian *Departamento de Ciências e Tecnologia*, *Conselho Nacional de Desenvolvimento Científico, Ministério da Saúde/Tecnologia de Insumos Estratégicos* (DECIT/CNPq/MS/SCTIE, process number 404277/2012-8).
