# Supplementary figures and images for: Genetic polymorphisms of the IL6 and NOD2 genes are risk factors for inflammatory reactions in leprosy
Source: PLoS Negl Trop Dis. 2017 Jul 17;11(7):e0005754. doi: 10.1371/journal.pntd.0005754 (PMC5531687; doi:10.1371/journal.pntd.0005754)

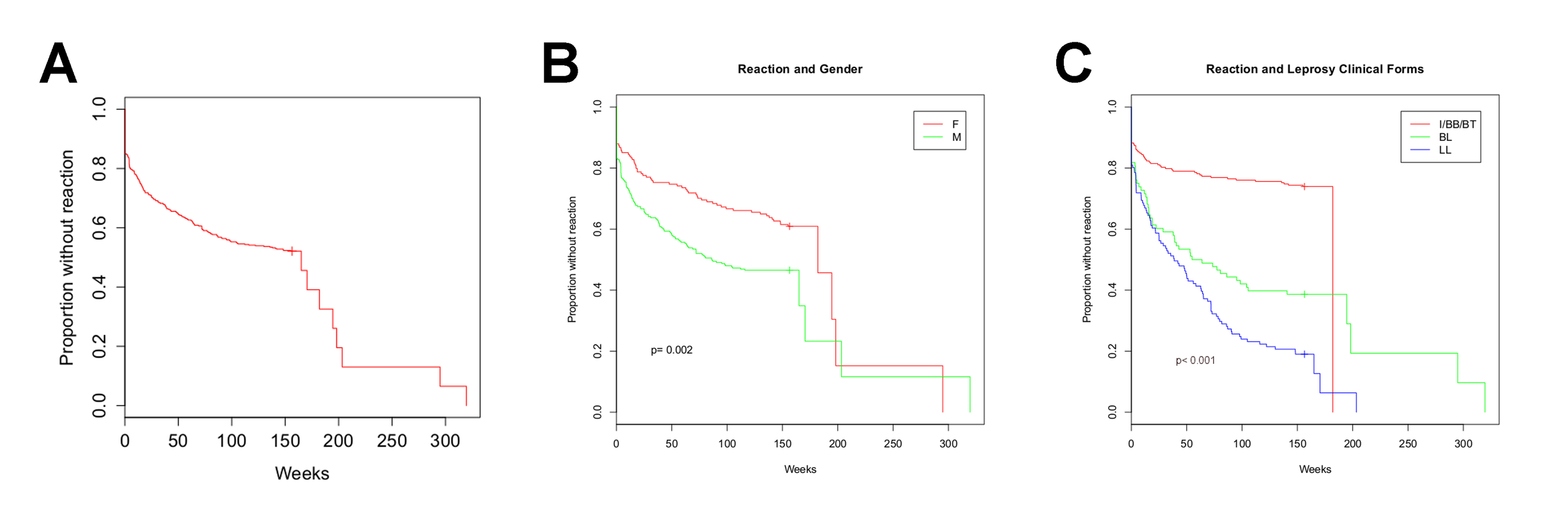

Supplement: S1 Fig — Cumulative proportion of reaction occurrence among leprosy patients overall (A), leprosy patients by gender (B), and by clinical forms (C). Kaplan–Meier curves were compared using a log-rank test. The + symbol indicates censured samples. P = p-value from a log-rank test. F = female, M = male, I = indeterminate leprosy, BT = borderline tuberculoid, BB = borderline borderline, BL = borderline lepromatous, LL = leprosy lepromatous. (TIF) [file pntd.0005754.s002.tif]

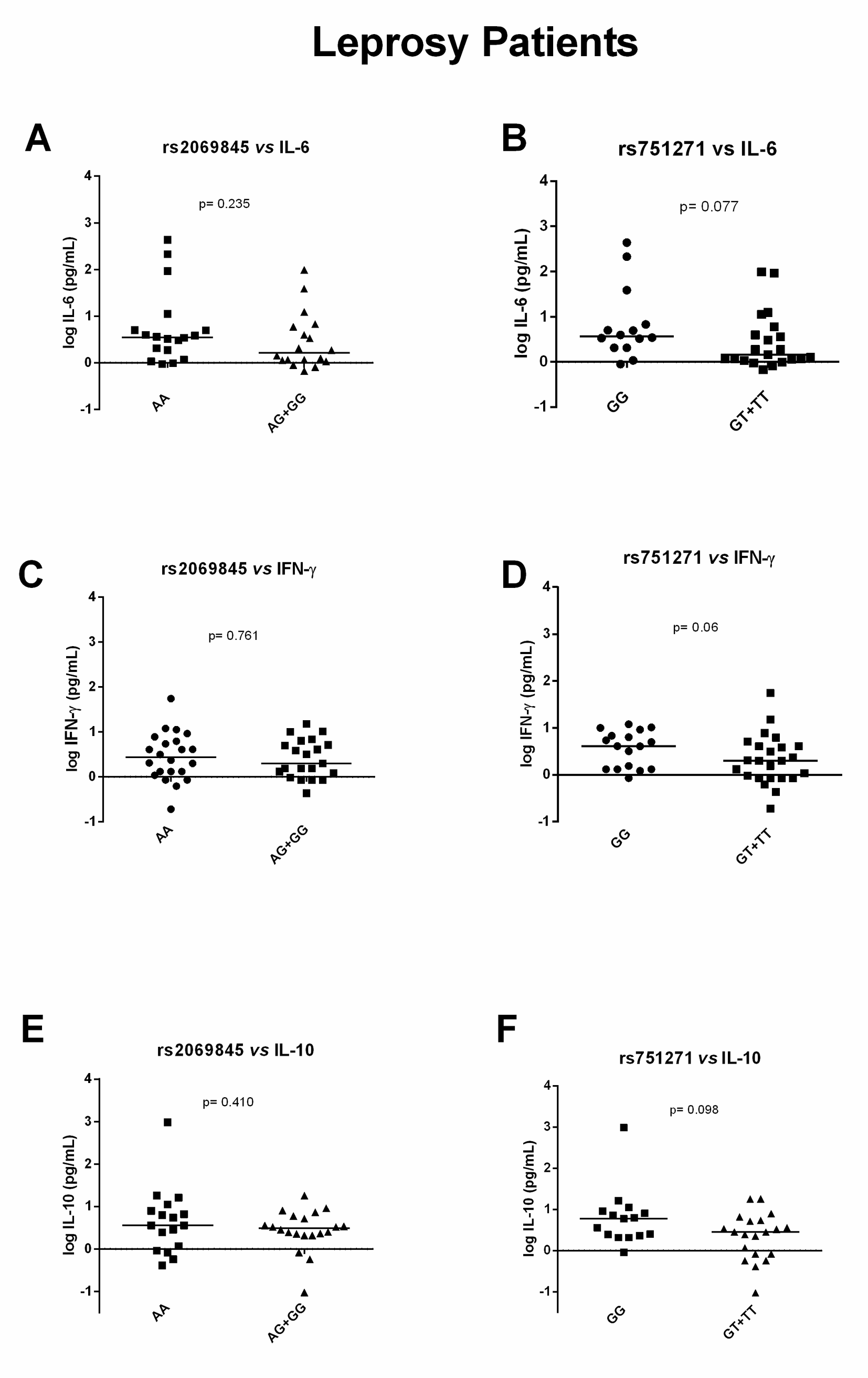

Supplement: S2 Fig — IL-6 (A and B), IFN-γ (C and D), and IL-10 (E and F) levels were quantified in the serum of leprosy patients using enzyme-linked immunosorbent assay (ELISA). Median dosage values were compared between groups by a Mann–Whitney t test. Sample size (N): A) AA = 18, AG+GG = 18; B) GG = 14, GT+TT = 21; C) AA = 22, AG+GG = 21; D) GG = 17, GT+TT = 21; E) AA = 17, AG+GG = 20; F) GG = 15; GT+TT = 20. (TIF) [file pntd.0005754.s003.tif]

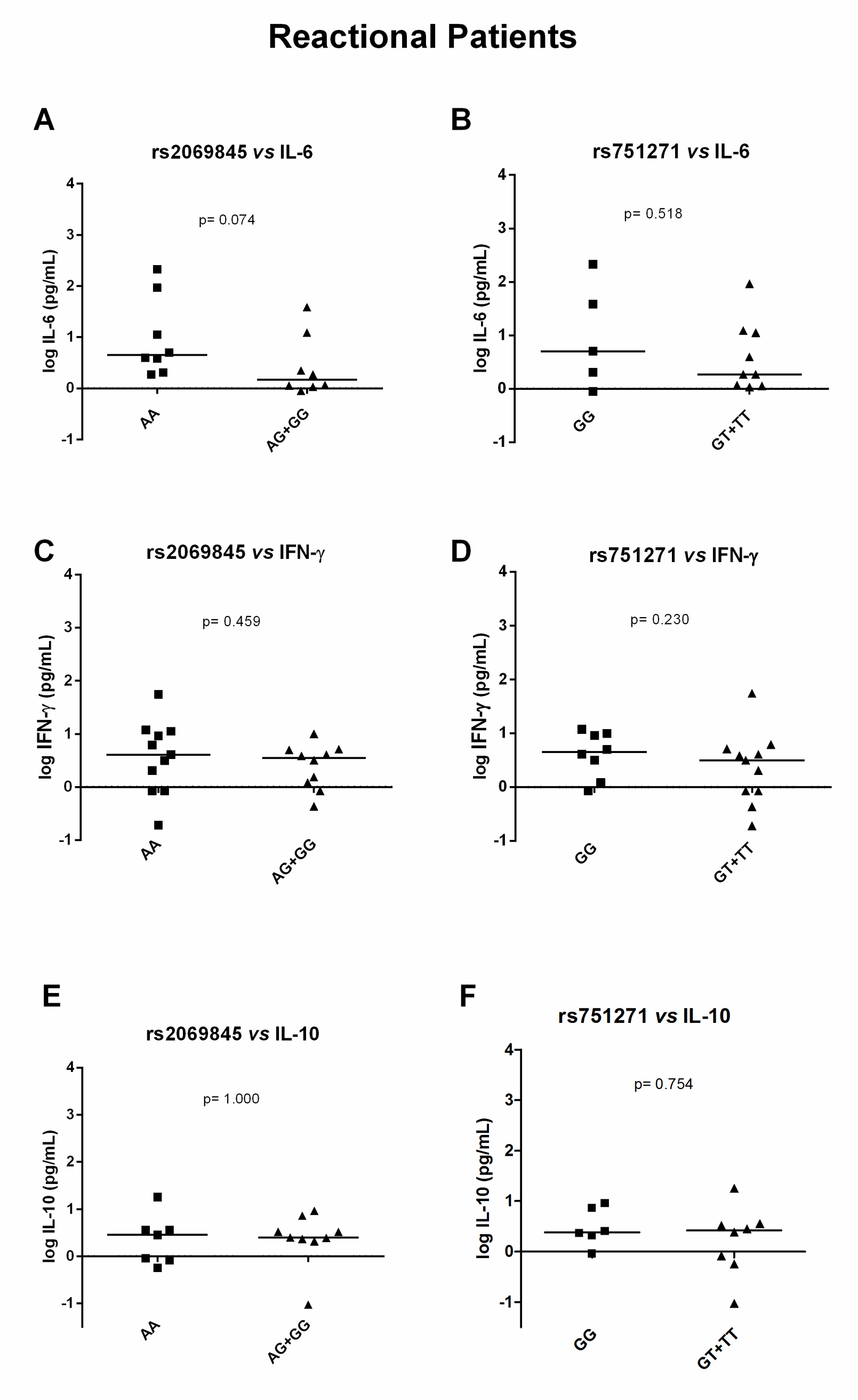

Supplement: S3 Fig — IL-6 (A and B), IFN-γ (C and D), and IL-10 (E and F) levels were quantified in the serum from reactional leprosy patients using enzyme-linked immunosorbent assay (ELISA). Median dosage values were compared between groups by a Mann–Whitney t test. Sample size (N): (A) AA = 8, AG+GG = 8; (B) GG = 5, GT+TT = 9; (C) AA = 11, AG+GG = 11; (D) GG = 9, GT+TT = 13; (E) AA = 7, AG+GG = 9; (F) GG = 6, GT+TT = 8. (TIF) [file pntd.0005754.s004.tif]

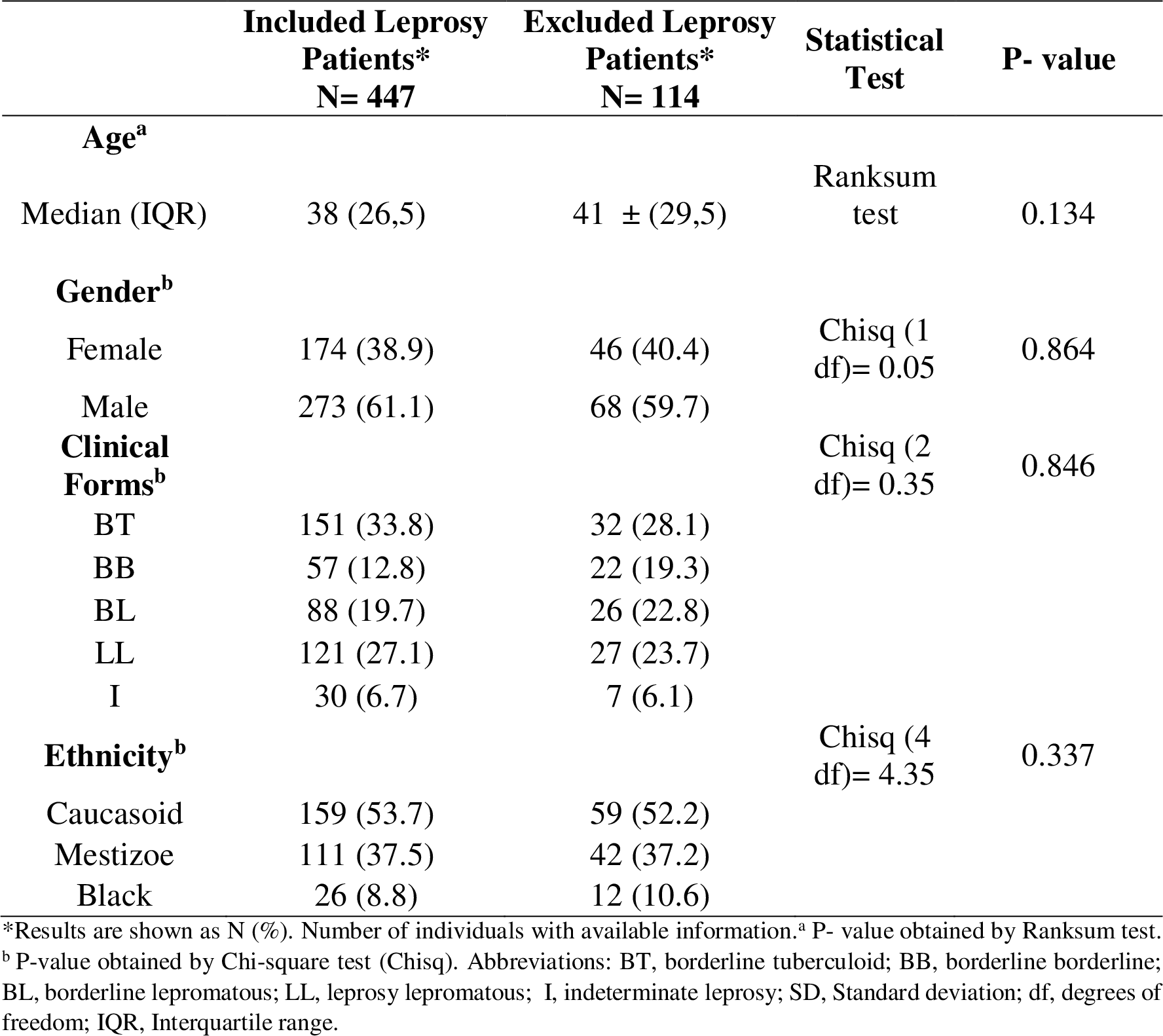

Supplement: S1 Table — (TIF) [file pntd.0005754.s005.tif]

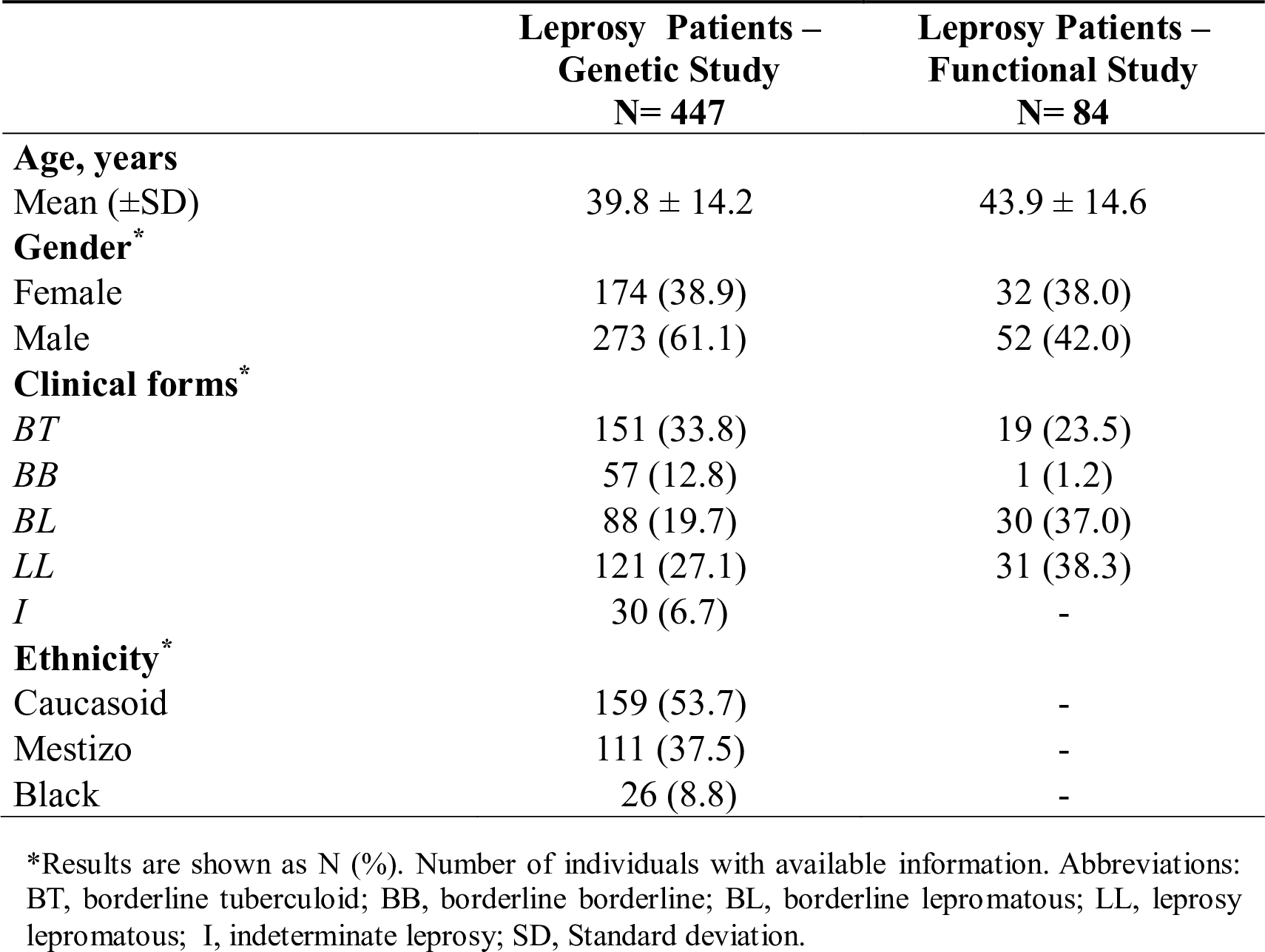

Supplement: S2 Table — (TIF) [file pntd.0005754.s006.tif]

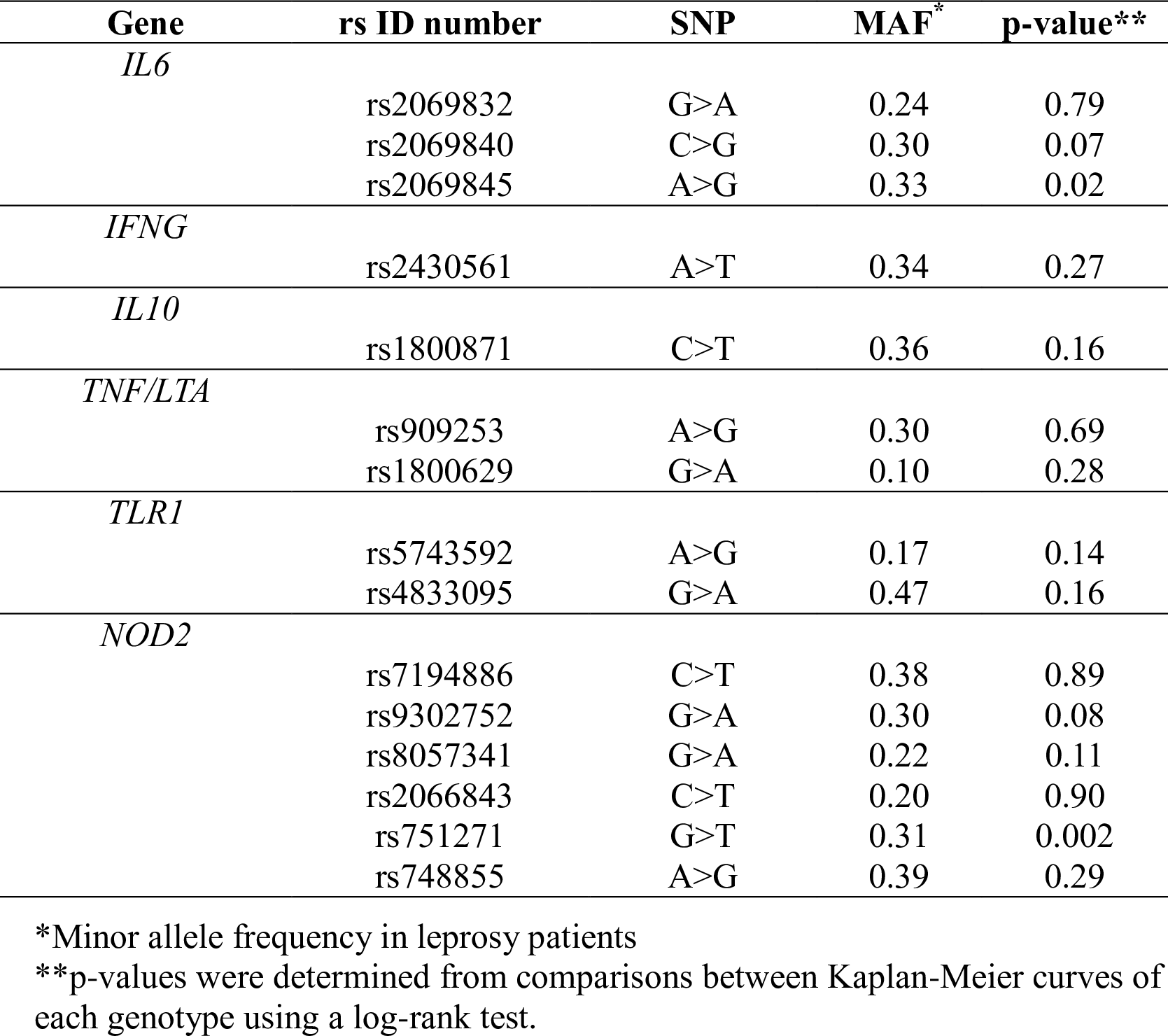

Supplement: S4 Table — (TIF) [file pntd.0005754.s008.tif]
