# Supplementary material for: Genetic polymorphisms of the IL6 and NOD2 genes are risk factors for inflammatory reactions in leprosy
Source: PLoS Negl Trop Dis. 2017 Jul 17;11(7):e0005754. doi: 10.1371/journal.pntd.0005754 (PMC5531687; doi:10.1371/journal.pntd.0005754)
Supplement: S3 Table — (PDF) [file pntd.0005754.s007.pdf]

	No Reaction		Reaction	
TNF rs1800629	**N**	**Freq**	**N**	**Freq**
G/G	167	83.5	169	84.9
G/A	27	13.5	29	14.6
A/A	6	3.0	1	0.5
A carriers	33	16.5	30	15.1
LTA rs909253				
A/A	84	49.4	72	46.2
A/G	71	41.8	67	42.9
G/G	15	8.8	17	10.9
G carriers	86	50.6	84	53.8
IL10 rs1800871				
C/C	72	40.9	60	35.3
C/T	83	47.2	79	46.5
T/T	21	11.9	31	18.2
T carriers	104	59.1	110	64.7
IFNG rs2430561				
A/A	80	44.9	95	51.6
A/T	76	42.7	68	37.0
T/T	22	12.4	21	11.4
T carriers	98	55.1	89	48.4
IL6 rs2069832				
G/G	85	57.0	89	61.8
G/A	56	37.6	49	34.0
A/A	8	5.4	6	4.2
A carriers	64	43.0	55	38.2
IL6 rs2069840				
C/C	72	48.0	79	58.1
C/G	64	42.7	53	39.0
G/G	14	9.3	4	2.9
G carriers	78	52.0	57	41.9
IL6 rs2069845				
A/A	72	45.3	48	32.7
A/G	69	43.4	80	54.4
G/G	18	11.3	19	12.9
G carriers	87	54.7	99	67.3
NOD2 rs751271				
G/G	72	44.7	71	47.3
G/T	80	49.7	55	36.7
T/T	9	5.6	24	16.0
T carriers	89	55.3	79	52.7
NOD2 rs2066843				
C/C	95	61.7	88	63.3

C/T	57	37.0	48	34.5
T/T	2	1.3	3	2.2
T carriers	59	38.3	51	36.7
NOD2 rs748855				
A/A	58	38.9	62	48.4
A/G	72	48.3	56	43.8
G/G	19	12.8	10	7.8
G carriers	91	61.1	66	51.6
NOD2 rs7194886				
C/C	60	37.5	61	41.2
C/T	80	50.0	71	48.0
T/T	20	12.5	16	10.8
T carriers	100	62.5	87	58.8
NOD2 rs9302752				
G/G	71	45.2	80	53.0
G/A	78	49.7	58	38.4
A/A	8	5.1	13	8.6
A carriers	86	54.8	71	47.0
NOD2 rs8057341				
G/G	112	60.2	110	57.3
G/A	66	35.5	66	34.4
A/A	8	4.3	16	8.3
A carriers	74	39.8	82	42.7
TLR1 rs5743592				
A/A	128	71.1	127	70.2
A/G	44	24.4	49	27.1
G/G	8	4.4	5	2.8
G carriers	52	28.9	54	29.8
TLR1 rs4833095				
G/G	62	33.3	57	30.2
G/A	74	39.8	90	47.6
A/A	50	26.9	42	22.2
A carriers	124	66.7	132	69.8

Abreviations : N= total count, Freq.= Frequency of each genotype/minor allele carrier
