# Supplementary material for: Genetic polymorphisms of the IL6 and NOD2 genes are risk factors for inflammatory reactions in leprosy
Source: PLoS Negl Trop Dis. 2017 Jul 17;11(7):e0005754. doi: 10.1371/journal.pntd.0005754 (PMC5531687; doi:10.1371/journal.pntd.0005754)
Supplement: S5 Table — (PDF) [file pntd.0005754.s009.pdf]

ID	Age	Gender*	Date of First Leprosy Diagnosis	Clinical Form	Reaction Status**	Date of First Reaction Episode	NOD2 rs751271	IL6 rs2069845
LEP001	51	M	October 10, 1995	LL	1	November 6, 1995	GT	AA
LEP002	20	M	March 6, 1987	BL	1	January 23, 1989	TT	GG
LEP003	59	F	June 2, 1997	BT	1	April 24, 1997	GG	AG
LEP004	53	M	March 4, 1996	LL	1	April 4, 1996	GT	AG
LEP005	20	M	August 28, 1996	BL	1	August 27, 1996	GT	AA
LEP006	21	M	September 20, 1991	BL	1	November 4, 1997	GG	AA
LEP007	25	M	June 28, 1990	LL	1	November 13, 1991	NA	NA
LEP008	54	F	October 9, 1997	BT	1	August 3, 1999	GG	AA
LEP009	24	M	September 17, 1996	LL	1	September 17, 1996	TT	AG
LEP010	60	M	February 27, 1997	BB	1	May 18, 1998	GT	AA
LEP011	23	M	August 18, 1998	LL	1	May 16, 1999	GG	AG
LEP012	50	M	January 7, 1997	BB	1	January 7, 1997	NA	NA
LEP013	15	M	December 8, 1994	BB	1	August 21, 1995	GT	AG
LEP014	39	M	July 21, 1998	LL	1	December 21, 1998	GT	AG
LEP015	14	M	November 28, 1996	BB	1	March 4, 1997	GG	AA
LEP016	21	M	September 1, 1998	LL	1	August 30, 1998	TT	AG
LEP017	18	M	April 6, 1992	BB	1	April 6, 1992	GT	AA
LEP018	31	F	August 19, 1997	I	1	March 23, 1998	GG	AG
LEP019	20	F	June 29, 1995	BL	1	March 10, 1998	TT	AA
LEP020	49	M	April 15, 1997	LL	1	July 6, 1999	TT	AG
LEP021	12	F	June 14, 1996	BL	1	November 10, 1997	GG	AG
LEP022	66	M	December 17, 1996	BL	1	January 15, 1997	GG	AG
LEP023	43	F	June 4, 1998	BB	1	December 10, 1998	GG	AG
LEP024	27	F	February 19, 1998	BB	1	January 27, 1998	NA	AG
LEP025	59	M	October 20, 1993	BL	1	December 20, 1993	GT	AG
LEP026	31	M	March 30, 1999	BT	1	May 3, 2000	NA	AG
LEP027	47	F	November 16, 1998	BT	1	January 4, 2000	TT	AG
LEP028	41	M	October 9, 1997	BB	1	October 9, 1997	GT	NA
LEP029	53	M	January 5, 1994	BL	1	November 1, 1994	GT	AG
LEP030	38	M	November 23, 1998	LL	1	November 23, 1998	GT	AA
LEP031	51	M	April 19, 1999	BB	1	April 19, 1999	TT	GG

LEP032	15	M	May 18, 1999	LL	1	June 13, 2000	NA	NA
LEP033	60	F	May 18, 1999	I	1	September 8, 1999	GT	AG
LEP034	66	M	September 28, 1992	BL	1	July 26, 1994	NA	AA
LEP035	56	F	December 10, 1998	LL	1	December 29, 1998	GG	GG
LEP036	21	F	February 22, 1999	BL	1	February 3, 1999	GT	AG
LEP037	46	F	February 3, 1992	LL	1	June 5, 1992	GT	AG
LEP038	41	F	January 8, 1998	LL	1	June 2, 1999	GT	AA
LEP039	59	M	February 28, 1991	BB	1	November 18, 1991	GG	AG
LEP040	51	F	May 5, 1995	BL	1	February 20, 1999	GT	AA
LEP041	31	M	June 22, 1999	LL	1	March 15, 2000	NA	NA
LEP042	41	M	July 15, 1999	BB	1	July 15, 1999	NA	NA
LEP043	52	M	December 14, 1988	LL	1	June 6, 1989	GG	GG
LEP044	37	M	June 27, 1995	LL	1	August 28, 1995	GG	AG
LEP045	50	F	November 10, 1988	BL	1	October 7, 1988	GT	AG
LEP046	29	M	July 22, 1996	BB	1	June 25, 1996	NA	NA
LEP047	73	M	November 3, 1998	BT	1	October 19, 1998	TT	AA
LEP048	38	M	March 9, 1988	LL	1	May 25, 1988	TT	NA
LEP049	64	F	July 22, 1999	LL	1	November 23, 2001	GT	AG
LEP050	58	F	February 16, 1987	BL	1	November 9, 1990	GG	AA
LEP051	27	F	September 13, 1999	LL	1	July 16, 2002	GT	GG
LEP052	41	F	October 1, 1998	BT	1	December 15, 1998	GG	AG
LEP053	25	M	November 18, 1999	LL	1	November 18, 1999	GT	NA
LEP054	42	M	November 11, 1999	BB	1	November 11, 1999	GT	GG
LEP055	28	M	December 14, 1999	LL	1	December 14, 1999	GT	AG
LEP056	42	M	January 3, 2000	LL	1	October 31, 2000	GT	GG
LEP057	35	M	June 22, 1999	BL	1	June 22, 1999	GT	AA
LEP058	9	M	January 12, 2000	BB	1	February 9, 2000	GG	AG
LEP059	57	M	January 31, 2000	BL	1	February 8, 2001	GT	AA
LEP060	26	M	January 9, 2001	LL	1	May 28, 2002	GG	AA
LEP061	30	M	December 16, 1999	LL	1	March 16, 2001	GG	GG
LEP062	23	M	March 14, 2000	LL	1	April 3, 2000	GG	AA
LEP063	18	M	April 3, 2000	LL	1	March 29, 2000	TT	GG
LEP064	22	F	March 20, 2000	BT	1	November 11, 2002	NA	NA

LEP065	16	M	April 4, 2000	BL	1	April 4, 2000	TT	AG
LEP066	61	M	March 27, 1995	LL	1	September 29, 1995	NA	NA
LEP067	42	M	May 3, 2000	LL	1	May 3, 2000	NA	NA
LEP068	46	M	March 20, 2000	BT	1	March 13, 2000	NA	NA
LEP069	76	M	May 22, 2000	BB	1	April 27, 2000	GG	GG
LEP070	56	M	June 7, 2000	LL	1	August 7, 2000	GG	AG
LEP071	30	M	January 3, 2000	LL	1	December 27, 1999	TT	AA
LEP072	49	F	January 8, 1998	BT	1	August 15, 2000	GG	AG
LEP073	28	F	October 5, 2000	LL	1	May 17, 2001	GG	AG
LEP074	45	M	October 10, 2000	LL	1	December 12, 2000	GT	GG
LEP075	24	F	September 18, 2000	LL	1	August 13, 2002	GG	AA
LEP076	30	F	April 1, 1999	BB	1	September 15, 1999	GG	AG
LEP077	48	F	October 25, 2000	BL	1	January 15, 2001	GT	AG
LEP078	22	M	October 24, 2000	LL	1	April 17, 2001	GT	AA
LEP079	12	M	December 1, 2000	BL	1	November 30, 2000	GG	AG
LEP080	16	M	November 1, 2000	BL	1	October 26, 2000	GG	AG
LEP081	56	M	November 23, 2000	BL	1	December 19, 2000	GT	NA
LEP082	20	M	November 21, 2000	BT	1	March 22, 2001	NA	NA
LEP083	61	M	January 9, 2001	BL	1	February 1, 2001	GG	AA
LEP084	38	F	November 30, 2000	LL	1	April 11, 2001	GT	AG
LEP085	41	F	November 22, 2000	LL	1	February 4, 2002	NA	NA
LEP086	61	M	February 21, 2001	BL	1	October 17, 2002	GT	AG
LEP087	40	F	April 24, 2001	BT	1	April 24, 2001	GT	AG
LEP088	35	M	May 2, 2001	BB	1	May 2, 2001	GG	AG
LEP089	64	F	May 22, 2001	BL	1	August 22, 2001	NA	NA
LEP090	18	F	July 4, 2001	BB	1	July 19, 2001	GG	AG
LEP091	72	M	July 9, 2001	LL	1	November 1, 2001	GT	AA
LEP092	47	F	July 1, 1999	BB	1	June 17, 1999	GG	AA
LEP093	50	F	August 6, 2001	BB	1	August 6, 2001	GG	AG
LEP094	24	M	October 9, 2001	LL	1	November 8, 2001	GT	AA
LEP095	14	M	October 8, 2001	LL	1	September 19, 2001	GG	AG
LEP096	60	F	November 21, 2001	BB	1	November 21, 2001	GG	AG
LEP097	29	F	November 14, 2001	BL	1	July 10, 2007	GT	GG

LEP098	33	M	January 22, 2002	BL	1	October 14, 2002	GG	AG
LEP099	34	M	February 19, 2002	BL	1	January 29, 2002	GG	AG
LEP100	53	M	January 15, 2002	BB	1	April 4, 2002	GG	AA
LEP101	37	F	January 7, 2002	BT	1	September 8, 2003	GT	AG
LEP102	74	M	March 12, 2002	BT	1	May 19, 2003	TT	AG
LEP103	16	M	March 11, 2002	BL	1	February 9, 2002	GG	AA
LEP104	50	M	April 8, 2002	BB	1	March 7, 2002	GG	AG
LEP105	41	M	April 25, 2002	LL	1	November 4, 2003	TT	AA
LEP106	9	F	May 20, 2002	BB	1	June 24, 2002	GT	AG
LEP107	22	M	June 4, 2002	BL	1	May 7, 2002	GG	AA
LEP108	11	M	April 22, 2002	BL	1	November 6, 2003	GT	AG
LEP109	30	M	July 18, 2002	LL	1	June 20, 2002	TT	AA
LEP110	36	M	September 2, 2002	LL	1	March 4, 2004	TT	GG
LEP111	23	M	August 22, 2002	LL	1	September 19, 2002	GG	GG
LEP112	26	F	November 19, 2002	BT	1	August 9, 2005	GG	AG
LEP113	58	F	July 3, 2002	BL	1	July 22, 2003	GG	AA
LEP114	21	M	September 30, 2002	BL	1	October 6, 2003	GG	AG
LEP115	28	F	September 18, 2002	LL	1	March 16, 2005	GG	AA
LEP116	37	M	September 30, 2002	BT	1	November 23, 2004	TT	AA
LEP117	58	M	November 5, 2002	BB	1	October 7, 2002	GT	AA
LEP118	51	M	December 17, 2002	BB	1	June 16, 2003	TT	AG
LEP119	60	F	February 6, 2003	BL	1	May 28, 2003	GT	AG
LEP120	26	M	March 11, 2003	BL	1	April 28, 2003	GT	AG
LEP121	56	M	September 9, 1985	BT	1	August 27, 1985	GG	AA
LEP122	37	M	April 14, 2003	LL	1	July 16, 2003	GG	AG
LEP123	56	M	April 7, 2003	BL	1	September 29, 2004	GG	AA
LEP124	39	M	March 25, 2003	LL	1	March 3, 2004	TT	GG
LEP125	49	M	May 5, 2003	BL	1	May 3, 2005	GT	AG
LEP126	35	F	April 28, 2003	BL	1	August 24, 2003	TT	AA
LEP127	55	F	May 8, 2003	BT	1	May 8, 2003	GG	AG
LEP128	45	F	January 15, 1988	BL	1	December 11, 1987	GG	AG
LEP129	24	M	July 12, 1999	LL	1	July 12, 1999	GT	GG
LEP130	66	F	September 1, 1987	BL	1	November 21, 1988	GT	AG

LEP131	55	F	October 14, 1999	BB	1	October 14, 1999	GG	AG
LEP132	46	F	May 26, 1999	BL	1	May 19, 1999	GT	AG
LEP133	32	F	October 21, 2003	BT	1	September 22, 2003	GG	AG
LEP134	18	M	January 28, 2004	LL	1	January 28, 2004	GG	AG
LEP135	20	M	July 3, 2003	LL	1	December 2, 2003	GT	AG
LEP136	28	M	September 18, 2003	LL	1	November 16, 2006	GT	NA
LEP137	49	F	July 17, 2003	BB	1	January 11, 2007	GT	NA
LEP138	23	M	September 2, 2003	LL	1	July 26, 2007	GG	NA
LEP139	46	M	September 17, 2003	LL	1	August 20, 2004	GG	GG
LEP140	29	M	October 16, 2003	BL	1	November 12, 2003	GT	AG
LEP141	59	M	October 9, 2003	LL	1	September 24, 2003	GG	NA
LEP142	59	M	October 20, 2003	BL	1	January 27, 2004	GG	NA
LEP143	26	F	October 14, 2003	BT	1	October 14, 2003	GG	AG
LEP144	33	M	October 16, 2003	LL	1	August 5, 2004	GT	AG
LEP145	22	F	June 2, 2004	LL	1	December 26, 2005	GG	AA
LEP146	28	F	May 27, 2004	LL	1	June 24, 2004	GG	AA
LEP147	33	M	June 17, 2004	LL	1	June 17, 2004	GG	AA
LEP148	27	M	December 29, 2003	LL	1	January 1, 2006	GG	AG
LEP149	21	M	November 24, 2003	LL	1	July 25, 2005	NA	AA
LEP150	55	M	October 3, 2002	BL	1	January 21, 2003	GG	AG
LEP151	73	M	February 17, 2004	BL	1	February 17, 2004	TT	NA
LEP152	34	M	July 29, 2003	BL	1	September 2, 2003	GG	AG
LEP153	63	F	November 5, 2003	LL	1	November 5, 2003	NA	AG
LEP154	49	F	June 28, 2004	BL	1	December 2, 2004	TT	AG
LEP155	24	M	August 17, 2004	LL	1	September 29, 2005	GG	AA
LEP156	40	F	April 12, 2005	LL	1	October 2, 2006	NA	GG
LEP157	75	F	April 11, 2005	BT	1	April 1, 2008	GT	AG
LEP158	20	M	December 5, 2004	LL	1	February 20, 2006	GG	AG
LEP159	56	M	June 10, 2005	LL	1	April 26, 2007	GG	AA
LEP160	47	M	September 20, 2004	BL	1	August 25, 2004	GG	AA
LEP161	40	F	June 23, 2005	LL	1	May 23, 2006	GT	AA
LEP162	68	M	December 14, 2004	LL	1	January 11, 2005	TT	AG
LEP163	29	M	June 14, 2005	BL	1	May 16, 2005	GG	AA

LEP164	36	F	October 5, 2004	LL	1	August 9, 2007	GT	AG
LEP165	29	M	June 20, 2005	LL	1	February 12, 2007	TT	AG
LEP166	47	M	July 4, 2005	BB	1	September 6, 2005	GT	AG
LEP167	53	M	September 14, 2004	BT	1	December 7, 2004	GG	AG
LEP168	66	M	June 30, 2005	BB	1	September 27, 2005	GG	GG
LEP169	41	F	June 21, 2005	LL	1	July 21, 2005	NA	AA
LEP170	45	M	November 8, 2005	LL	1	March 5, 2007	NA	NA
LEP171	31	M	September 6, 2005	LL	1	April 11, 2006	NA	NA
LEP172	22	M	September 29, 2005	BB	1	October 17, 2005	NA	NA
LEP173	43	M	August 22, 2005	BL	1	January 2, 2006	NA	NA
LEP174	48	M	September 26, 2005	BL	1	October 24, 2005	NA	NA
LEP175	23	M	August 22, 2005	LL	1	August 30, 2005	NA	NA
LEP176	28	M	October 20, 2005	LL	1	June 26, 2006	NA	NA
LEP177	25	F	November 23, 2005	LL	1	July 13, 2006	NA	NA
LEP178	41	M	November 7, 2005	LL	1	February 12, 2009	NA	NA
LEP179	54	M	December 15, 2005	LL	1	March 9, 2006	NA	NA
LEP180	38	M	December 1, 2005	BL	1	September 11, 2006	NA	NA
LEP181	20	M	May 4, 2006	LL	1	November 22, 2006	NA	NA
LEP182	24	M	January 26, 2006	LL	1	June 14, 2007	NA	NA
LEP183	65	F	March 9, 2006	LL	1	July 11, 2006	NA	NA
LEP184	17	M	March 23, 2006	BL	1	March 23, 2006	NA	NA
LEP185	34	F	June 22, 2006	LL	1	June 22, 2006	NA	NA
LEP186	49	M	March 14, 2006	LL	1	June 19, 2006	NA	NA
LEP187	54	M	January 15, 2007	BT	1	December 11, 2006	NA	NA
LEP188	28	M	September 19, 2006	LL	1	January 2, 2007	NA	NA
LEP189	79	M	November 6, 2006	BL	1	August 7, 2007	NA	NA
LEP190	30	M	August 7, 2006	LL	1	August 7, 2006	NA	NA
LEP191	10	M	September 11, 2006	BT	1	September 11, 2006	NA	NA
LEP192	22	M	October 17, 2006	LL	1	March 4, 2008	NA	NA
LEP193	42	M	August 31, 2006	BT	1	August 31, 2006	NA	NA
LEP194	25	F	November 30, 2006	BL	1	December 8, 2008	NA	NA
LEP195	30	M	August 10, 2006	BL	1	February 27, 2007	NA	NA
LEP196	44	M	August 15, 2006	LL	1	February 6, 2007	NA	NA

LEP197	21	M	January 17, 2006	LL	1	April 12, 2007	NA	NA
LEP198	70	F	April 9, 2007	LL	1	April 2, 2007	NA	NA
LEP199	52	F	April 26, 2007	BL	1	September 5, 2007	NA	NA
LEP200	68	F	May 7, 2007	BL	1	August 15, 2007	NA	NA
LEP201	48	M	February 27, 2007	BL	1	November 26, 2007	NA	NA
LEP202	38	M	December 18, 2006	LL	1	December 12, 2007	NA	NA
LEP203	16	F	January 18, 2007	LL	1	January 11, 2007	NA	NA
LEP204	26	M	July 17, 2007	LL	1	November 13, 2007	NA	NA
LEP205	20	M	June 26, 2007	LL	1	June 26, 2007	NA	NA
LEP206	28	M	August 28, 2007	BT	1	August 7, 2007	NA	NA
LEP207	66	M	September 4, 2007	LL	1	May 28, 2009	NA	NA
LEP208	25	F	September 25, 2007	LL	1	June 22, 2009	NA	NA
LEP209	26	F	October 23, 2007	LL	1	October 23, 2007	NA	NA
LEP210	59	M	October 24, 2007	BB	1	November 21, 2007	NA	NA
LEP211	53	F	October 22, 2004	BT	1	March 10, 2006	NA	NA
LEP212	19	M	January 7, 2008	BT	1	February 18, 2008	NA	NA
LEP213	19	M	March 3, 2008	LL	1	April 1, 2008	NA	NA
LEP214	70	F	April 3, 2008	LL	1	July 2, 2009	NA	NA
LEP215	41	M	June 16, 2008	LL	1	June 2, 2009	NA	NA
LEP216	25	M	June 24, 2008	LL	1	June 8, 2009	NA	NA
LEP217	71	M	July 2, 2008	BL	1	October 16, 2008	NA	NA
LEP218	37	F	August 4, 2008	LL	1	July 18, 2008	NA	NA
LEP219	33	M	August 7, 2008	BT	1	September 4, 2008	NA	NA
LEP220	21	M	December 6, 2005	LL	1	February 15, 2006	NA	NA
LEP221	27	M	July 13, 2006	BB	1	September 4, 2006	NA	NA
LEP222	27	F	May 21, 2007	LL	1	May 21, 2007	NA	NA
LEP223	59	F	September 4, 1997	BT	0	NA	GG	AG
LEP224	50	F	July 9, 1996	BB	0	NA	GG	AG
LEP225	10	F	March 26, 1998	BB	0	NA	GT	AA
LEP226	49	M	January 2, 1992	BL	0	NA	GT	AG
LEP227	25	F	August 31, 1998	BT	0	NA	GT	GG
LEP228	66	M	December 9, 1998	BT	0	NA	GT	AG
LEP229	19	F	March 16, 1998	I	0	NA	GT	AG

LEP230	57	M	December 4, 1997	BT	0	NA	GT	AG
LEP231	33	M	February 27, 1987	BL	0	NA	GT	AG
LEP232	16	M	March 9, 1988	BL	0	NA	GG	AG
LEP233	73	F	August 26, 1998	BT	0	NA	GT	GG
LEP234	40	F	August 24, 1998	BT	0	NA	GT	AG
LEP235	22	F	March 25, 1987	I	0	NA	GT	AA
LEP236	66	M	September 24, 1998	BL	0	NA	GT	AA
LEP237	36	M	June 14, 1994	BB	0	NA	GG	AA
LEP238	30	M	October 2, 1996	BL	0	NA	NA	AA
LEP239	14	M	October 29, 1998	BT	0	NA	NA	NA
LEP240	26	M	July 9, 1990	LL	0	NA	NA	AG
LEP241	40	F	August 19, 1998	BT	0	NA	NA	AA
LEP242	45	M	January 15, 1986	BL	0	NA	NA	NA
LEP243	22	F	December 16, 1988	LL	0	NA	NA	NA
LEP244	45	M	March 1, 1994	LL	0	NA	NA	NA
LEP245	18	F	April 20, 1999	BT	0	NA	GT	AG
LEP246	24	M	February 25, 1999	BT	0	NA	GG	AG
LEP247	3	M	February 9, 1999	BL	0	NA	GG	AG
LEP248	25	M	January 11, 1999	I	0	NA	GT	AG
LEP249	69	M	October 19, 1992	BL	0	NA	TT	AA
LEP250	36	F	June 14, 1999	I	0	NA	NA	NA
LEP251	18	M	November 10, 1998	BT	0	NA	GG	AA
LEP252	29	M	February 8, 1999	BT	0	NA	GG	AA
LEP253	31	M	March 30, 1999	BB	0	NA	NA	NA
LEP254	52	F	February 9, 1999	BB	0	NA	GG	AA
LEP255	55	M	February 25, 1999	I	0	NA	NA	NA
LEP256	8	M	June 14, 1999	BT	0	NA	GT	AA
LEP257	67	M	January 26, 1999	BT	0	NA	GT	AG
LEP258	50	M	March 20, 1997	BT	0	NA	NA	NA
LEP259	29	F	August 13, 1997	BT	0	NA	TT	AA
LEP260	46	M	June 17, 1997	BT	0	NA	GT	GG
LEP261	72	F	July 15, 1999	I	0	NA	GG	AA
LEP262	7	F	February 18, 1999	BT	0	NA	GG	AA

LEP263	31	M	April 14, 1989	BL	0	NA	GT	AA
LEP264	43	M	October 14, 1988	BL	0	NA	GT	AG
LEP265	14	F	July 22, 1999	BT	0	NA	GG	AA
LEP266	40	M	February 2, 1999	BT	0	NA	GT	AG
LEP267	81	M	August 17, 1999	BT	0	NA	GT	AA
LEP268	28	M	December 15, 1987	LL	0	NA	GT	AA
LEP269	60	M	July 29, 1999	I	0	NA	GG	AG
LEP270	55	F	June 22, 1998	BT	0	NA	GT	AA
LEP271	57	F	August 31, 1993	BT	0	NA	NA	NA
LEP272	31	F	January 13, 1992	BT	0	NA	NA	NA
LEP273	41	M	July 6, 1998	BT	0	NA	GG	AA
LEP274	39	M	October 1, 1990	LL	0	NA	NA	NA
LEP275	35	M	August 1, 1999	BT	0	NA	GG	AA
LEP276	14	M	September 12, 1989	BL	0	NA	GG	AG
LEP277	38	M	September 1, 1999	LL	0	NA	GT	NA
LEP278	50	F	August 30, 1999	BT	0	NA	GG	AG
LEP279	23	F	July 1, 1999	I	0	NA	GG	GG
LEP280	34	M	August 18, 1987	BL	0	NA	GG	AG
LEP281	27	M	October 18, 1999	BT	0	NA	GG	AA
LEP282	65	M	August 11, 1987	LL	0	NA	GG	GG
LEP283	31	F	October 21, 1999	I	0	NA	GT	AG
LEP284	50	M	July 5, 2001	BB	0	NA	GT	AA
LEP285	22	F	December 16, 1999	BT	0	NA	GG	AA
LEP286	50	F	December 1, 1999	BT	0	NA	GG	GG
LEP287	30	M	January 17, 2000	BT	0	NA	GG	AG
LEP288	38	F	May 18, 1998	I	0	NA	GG	AA
LEP289	57	M	November 30, 1999	BB	0	NA	NA	NA
LEP290	24	M	April 13, 2000	I	0	NA	NA	NA
LEP291	74	M	March 14, 2000	LL	0	NA	NA	NA
LEP292	15	M	July 3, 2000	BT	0	NA	GG	AA
LEP293	18	M	July 25, 2000	BT	0	NA	GG	AA
LEP294	16	F	June 27, 2000	BT	0	NA	GT	AA
LEP295	20	F	June 26, 2000	BT	0	NA	GT	AA

LEP296	50	M	April 27, 2000	BB	0	NA	GG	AG
LEP297	40	M	August 8, 2000	BB	0	NA	GG	AA
LEP298	68	F	March 20, 2000	BL	0	NA	GT	AA
LEP299	62	F	July 20, 2000	BT	0	NA	GG	AA
LEP300	69	F	August 28, 2000	BT	0	NA	TT	AG
LEP301	57	M	September 18, 2000	BT	0	NA	GT	AG
LEP302	22	M	November 1, 2000	BT	0	NA	TT	AG
LEP303	17	M	September 4, 2000	BT	0	NA	GG	AA
LEP304	69	F	October 16, 2000	BT	0	NA	GT	AG
LEP305	62	M	November 7, 2000	BT	0	NA	NA	NA
LEP306	37	M	October 17, 2000	BT	0	NA	GT	AG
LEP307	17	F	January 21, 1998	BT	0	NA	GT	AG
LEP308	61	F	December 12, 2000	BT	0	NA	NA	NA
LEP309	41	M	February 12, 2001	BT	0	NA	TT	GG
LEP310	32	M	January 29, 2001	BL	0	NA	GG	AG
LEP311	53	F	December 14, 2000	BT	0	NA	TT	AA
LEP312	42	M	August 29, 2000	LL	0	NA	GT	AA
LEP313	57	M	October 4, 2000	BT	0	NA	GG	AG
LEP314	54	M	August 31, 2000	BB	0	NA	TT	AG
LEP315	33	M	June 12, 2000	BB	0	NA	GG	AA
LEP316	16	M	August 5, 2001	I	0	NA	TT	AA
LEP317	53	M	June 6, 2001	BB	0	NA	GG	AG
LEP318	64	M	May 4, 2001	BL	0	NA	GT	AG
LEP319	35	M	June 25, 2001	BT	0	NA	GT	AA
LEP320	22	M	July 17, 2001	BT	0	NA	TT	AA
LEP321	39	F	August 16, 2001	I	0	NA	GG	AA
LEP322	36	M	July 23, 2001	BT	0	NA	GT	AG
LEP323	16	F	July 25, 2001	I	0	NA	GT	AG
LEP324	34	F	July 12, 2001	BT	0	NA	GG	AA
LEP325	20	F	August 6, 2001	BB	0	NA	GT	AA
LEP326	29	F	September 26, 2000	BT	0	NA	GG	GG
LEP327	30	M	August 6, 2001	BT	0	NA	GT	AA
LEP328	24	F	October 19, 2000	BT	0	NA	GG	AG

LEP329	68	F	September 27, 2001	BT	0	NA	GG	GG
LEP330	62	F	September 27, 2001	BT	0	NA	GT	AG
LEP331	42	M	November 22, 2001	I	0	NA	GT	AA
LEP332	47	F	December 11, 2001	BT	0	NA	GT	AA
LEP333	32	F	March 21, 1990	I	0	NA	GT	AG
LEP334	26	F	November 12, 2001	BT	0	NA	GT	AG
LEP335	25	F	January 15, 2002	BT	0	NA	GT	AA
LEP336	45	F	February 12, 2002	BT	0	NA	GT	AA
LEP337	53	F	February 19, 2002	BT	0	NA	GG	AG
LEP338	65	M	April 22, 2002	BT	0	NA	GG	AG
LEP339	11	M	May 8, 2002	BB	0	NA	GT	AG
LEP340	32	F	May 23, 2002	I	0	NA	GG	AA
LEP341	62	M	June 10, 2002	BT	0	NA	GT	AG
LEP342	10	F	June 18, 2002	BT	0	NA	GT	AG
LEP343	36	M	June 27, 2002	BL	0	NA	GG	AG
LEP344	51	F	August 27, 2002	BB	0	NA	GT	GG
LEP345	47	F	July 22, 2002	BT	0	NA	GG	AG
LEP346	30	M	June 25, 2002	BT	0	NA	GG	GG
LEP347	6	F	May 25, 2002	BT	0	NA	GG	AA
LEP348	32	F	January 28, 2003	BT	0	NA	GG	AA
LEP349	78	F	August 22, 2002	BT	0	NA	GT	AG
LEP350	32	M	September 25, 2002	I	0	NA	GT	AG
LEP351	60	F	September 19, 2002	BT	0	NA	GG	AA
LEP352	37	F	July 22, 2002	BT	0	NA	GG	AA
LEP353	23	F	October 8, 2002	BT	0	NA	GG	NA
LEP354	48	F	October 1, 2002	BT	0	NA	GG	AA
LEP355	45	M	October 31, 2002	BT	0	NA	GT	AG
LEP356	25	M	October 21, 2002	LL	0	NA	GT	AA
LEP357	62	F	October 15, 2002	I	0	NA	GG	AG
LEP358	14	F	October 21, 2002	BL	0	NA	GT	AG
LEP359	24	M	November 19, 2002	BL	0	NA	GG	AA
LEP360	53	F	December 26, 2002	BT	0	NA	GT	GG
LEP361	35	F	December 10, 2002	BT	0	NA	GT	AG

LEP362	36	F	February 18, 2003	BL	0	NA	GG	AA
LEP363	56	F	March 24, 2003	BT	0	NA	GT	AG
LEP364	5	M	July 28, 1988	BT	0	NA	GG	AG
LEP365	29	M	May 8, 2003	LL	0	NA	GG	AA
LEP366	28	F	May 19, 2003	I	0	NA	GG	AA
LEP367	54	F	June 23, 2003	I	0	NA	GG	AA
LEP368	53	F	June 25, 2003	BT	0	NA	GT	AA
LEP369	54	M	May 12, 2003	I	0	NA	GG	AA
LEP370	48	M	October 28, 2003	BT	0	NA	GG	AG
LEP371	51	M	January 19, 2004	BT	0	NA	GT	GG
LEP372	51	M	November 24, 2003	BL	0	NA	GT	AG
LEP373	40	M	January 13, 2004	BB	0	NA	GG	AA
LEP374	25	F	August 20, 2003	BT	0	NA	GT	NA
LEP375	22	M	September 30, 2003	I	0	NA	GT	AA
LEP376	42	F	July 16, 2003	BT	0	NA	GT	AG
LEP377	13	M	September 15, 2003	BT	0	NA	GT	GG
LEP378	34	M	July 14, 2003	BL	0	NA	GT	NA
LEP379	40	F	September 4, 2003	BT	0	NA	GT	NA
LEP380	11	F	August 4, 2003	BT	0	NA	GT	NA
LEP381	41	F	October 6, 2003	BT	0	NA	GG	GG
LEP382	53	F	August 21, 2003	BL	0	NA	GT	AG
LEP383	27	F	November 4, 2003	BT	0	NA	GG	GG
LEP384	46	F	November 13, 2003	BT	0	NA	GT	NA
LEP385	47	M	November 10, 2003	BT	0	NA	GG	GG
LEP386	26	F	May 8, 2004	BT	0	NA	GG	AG
LEP387	49	F	July 3, 2003	BT	0	NA	NA	NA
LEP388	52	F	December 1, 2003	BT	0	NA	GG	AA
LEP389	67	F	April 27, 2004	BT	0	NA	GG	AA
LEP390	35	M	August 7, 2003	BT	0	NA	GG	AA
LEP391	25	F	April 15, 2004	BL	0	NA	GT	AA
LEP392	54	M	March 22, 2004	BT	0	NA	GT	AG
LEP393	46	M	May 10, 2005	BT	0	NA	GG	AG
LEP394	49	F	June 13, 2005	BT	0	NA	NA	NA

LEP395	68	M	September 13, 2004	BL	0	NA	GT	AA
LEP396	39	M	September 8, 2004	LL	0	NA	GT	AA
LEP397	54	M	September 9, 2004	LL	0	NA	GT	AA
LEP398	26	M	September 30, 2004	I	0	NA	GT	AA
LEP399	56	M	September 30, 2004	BT	0	NA	GG	AG
LEP400	46	M	August 25, 2004	BT	0	NA	NA	AG
LEP401	56	F	November 16, 2004	BT	0	NA	GT	AG
LEP402	21	M	June 30, 2005	BT	0	NA	GT	AG
LEP403	60	F	April 28, 2005	BT	0	NA	GG	AG
LEP404	12	F	July 5, 2005	I	0	NA	GT	GG
LEP405	55	M	July 11, 2005	BT	0	NA	GT	AG
LEP406	45	M	October 19, 2004	BT	0	NA	NA	AA
LEP407	41	F	November 16, 2005	I	0	NA	NA	NA
LEP408	66	F	September 22, 2005	BT	0	NA	NA	NA
LEP409	48	F	September 20, 2005	BT	0	NA	NA	NA
LEP410	59	F	March 21, 2005	BT	0	NA	NA	NA
LEP411	46	M	April 26, 2005	BB	0	NA	NA	NA
LEP412	38	M	October 25, 2005	LL	0	NA	NA	NA
LEP413	38	M	November 8, 2005	BB	0	NA	NA	NA
LEP414	35	M	March 21, 2006	LL	0	NA	NA	NA
LEP415	37	F	February 7, 2006	BT	0	NA	NA	NA
LEP416	64	M	May 25, 2006	BT	0	NA	NA	NA
LEP417	62	M	March 14, 2006	LL	0	NA	NA	NA
LEP418	56	F	March 7, 2006	I	0	NA	NA	NA
LEP419	36	M	March 28, 2006	BL	0	NA	NA	NA
LEP420	27	M	February 8, 2006	LL	0	NA	NA	NA
LEP421	51	M	March 14, 2006	BB	0	NA	NA	NA
LEP422	52	F	December 11, 2006	LL	0	NA	NA	NA
LEP423	64	M	January 9, 2007	LL	0	NA	NA	NA
LEP424	35	M	September 18, 2006	BT	0	NA	NA	NA
LEP425	54	F	October 30, 2006	BT	0	NA	NA	NA
LEP426	41	F	March 22, 2007	BT	0	NA	NA	NA
LEP427	39	M	April 25, 2007	BL	0	NA	NA	NA

LEP428	57	M	February 27, 2007	BL	0	NA	NA	NA
LEP429	23	F	April 3, 2007	BL	0	NA	NA	NA
LEP430	29	M	May 8, 2007	BT	0	NA	NA	NA
LEP431	60	M	October 29, 2007	BT	0	NA	NA	NA
LEP432	53	F	June 19, 2007	BT	0	NA	NA	NA
LEP433	36	M	August 13, 2007	BT	0	NA	NA	NA
LEP434	18	M	May 25, 2007	I	0	NA	NA	NA
LEP435	26	M	October 10, 2007	BB	0	NA	NA	NA
LEP436	52	M	November 27, 2007	BL	0	NA	NA	NA
LEP437	58	M	February 14, 2008	BL	0	NA	NA	NA
LEP438	51	F	March 3, 2008	BT	0	NA	NA	NA
LEP439	67	M	April 1, 2008	BB	0	NA	NA	NA
LEP440	41	M	April 3, 2008	BB	0	NA	NA	NA
LEP441	28	F	April 7, 2008	BT	0	NA	NA	NA
LEP442	54	M	April 9, 2008	BB	0	NA	NA	NA
LEP443	45	F	June 2, 2008	BT	0	NA	NA	NA
LEP444	40	M	July 23, 2008	BB	0	NA	NA	NA
LEP445	46	F	September 2, 2008	BT	0	NA	NA	NA
LEP446	61	M	August 7, 2008	LL	0	NA	NA	NA
LEP447	60	F	November 27, 2006	BT	0	NA	NA	NA

* Gender: F= Female; M= Male

** Reaction status: 1= reaction; 0= no reaction
