# Supplementary material for: Genetic polymorphisms of the IL6 and NOD2 genes are risk factors for inflammatory reactions in leprosy
Source: PLoS Negl Trop Dis. 2017 Jul 17;11(7):e0005754. doi: 10.1371/journal.pntd.0005754 (PMC5531687; doi:10.1371/journal.pntd.0005754)
Supplement: S6 Table — (PDF) [file pntd.0005754.s010.pdf]

ID	Gender*	Age	Clinical Form	Reaction Status**	NOD2 rs751271	IL6 rs2069845	IL-6 (pg/mL)	IFN-γ (pg/mL)	IL-10 (pg/mL)
LEPFUN001	F	42	BL	1	NA	NA	1.25	1.54	0.99
LEPFUN002	M	27	LL	1	NA	NA	513.36	21.60	0.09
LEPFUN003	F	43	NA	1	GT	AA	1.87	0.85	0.82
LEPFUN004	M	35	BL	1	NA	NA	0.80	1.31	0.03
LEPFUN005	M	27	LL	1	GG	AA	5.01	11.91	0
LEPFUN006	F	69	LL	1	NA	NA	4.47	3.04	0
LEPFUN007	M	19	LL	1	NA	NA	116.08	23.31	4.70
LEPFUN008	F	41	BL	1	NA	AG	NA	1.54	3.29
LEPFUN009	M	42	BL	1	NA	NA	0.08	3.19	0.09
LEPFUN010	M	59	BL	1	NA	NA	0	0.19	0
LEPFUN011	F	60	LL	1	GG	AA	0	9.18	0
LEPFUN012	F	32	BL	1	NA	AA	3.82	11.20	3.58
LEPFUN013	F	62	NA	1	NA	NA	4.85	7.51	6.57
LEPFUN014	M	62	BL	1	NA	NA	17.28	5.58	0
LEPFUN015	F	46	BL	1	NA	NA	0	0	0
LEPFUN016	M	61	LL	1	GT	AA	92.41	0.19	0.57
LEPFUN017	M	39	LL	1	NA	NA	412.01	NA	NA
LEPFUN018	M	12	BL	1	NA	NA	0	18.55	5.16
LEPFUN019	M	36	LL	1	NA	NA	0	0	0
LEPFUN020	F	57	BL	1	NA	NA	2.74	21.79	0
LEPFUN021	F	53	BL	1	NA	NA	0	0	0
LEPFUN022	M	48	LL	1	NA	NA	54.26	7.44	0
LEPFUN023	M	68	LL	1	GT	AG	0	0.85	0
LEPFUN024	M	68	LL	1	GT	AG	12.31	5.13	0.09
LEPFUN025	F	50	BL	1	GG	AG	0	9.98	2.31
LEPFUN026	F	61	LL	1	GT	AG	1.13	0.43	0
LEPFUN027	M	21	LL	1	GG	AA	213.44	4.08	0
LEPFUN028	M	63	BL	1	TT	AG	1.07	4.06	2.44
LEPFUN029	F	71	BB	1	GG	AG	0	1.21	7.28
LEPFUN030	M	64	BL	1	GG	AA	2.04	0.85	0.91
LEPFUN031	M	34	BL	1	TT	AA	0	6.20	18.04
LEPFUN032	M	64	LL	1	GG	AG	38.66	3.19	9.10
LEPFUN033	M	21	LL	1	GG	AG	0	0	2.52
LEPFUN034	F	30	BL	1	GT	AG	1.87	3.84	3.30
LEPFUN035	M	59	BL	1	GT	AA	3.95	3.15	3.58
LEPFUN036	M	20	LL	1	GT	AA	0	2.04	2.83
LEPFUN037	F	18	BT	1	GG	AG	0.89	4.98	2.07
LEPFUN038	M	68	NA	1	GT	AA	11.16	55.13	0
LEPFUN039	F	45	BL	1	NA	NA	22.40	2.32	0
LEPFUN040	F	44	LL	0	NA	NA	1.16	5.44	2.72
LEPFUN041	M	45	BL	0	NA	NA	1.69	1.65	10.32
LEPFUN042	M	26	BL	0	NA	NA	1.25	2.23	9.05
LEPFUN043	M	51	BL	0	GT	AA	0.98	2.00	0.41
LEPFUN044	M	60	BT	0	NA	NA	1.07	1.77	1.08

LEPFUN045	F	62	BT	0	NA	NA	1.34	1.31	1.26
LEPFUN046	M	37	LL	0	NA	NA	0.98	0.62	4.76
LEPFUN047	M	45	BT	0	NA	NA	2.13	1.54	5.76
LEPFUN048	F	12	BT	0	NA	NA	4.55	2.23	5.56
LEPFUN049	F	12	BT	0	NA	NA	0.80	0.85	1.17
LEPFUN050	M	25	BL	0	GG	AG	2.04	1.54	8.01
LEPFUN051	M	60	BT	0	GT	AG	1.16	0.85	0.57
LEPFUN052	M	68	LL	0	NA	NA	1.16	0.85	0
LEPFUN053	F	61	LL	0	GT	AG	0.80	1.31	3.39
LEPFUN054	F	57	BT	0	GG	AA	432.98	1.31	968.13
LEPFUN055	M	58	BL	0	GT	GG	5.92	NA	2.26
LEPFUN056	M	47	LL	0	GG	AG	3.35	6.82	2.07
LEPFUN057	F	31	LL	0	NA	NA	55.52	7.74	11.81
LEPFUN058	F	31	LL	0	GG	AA	1.07	5.44	2.44
LEPFUN059	M	16	BT	0	GT	AG	1.25	1.54	5.16
LEPFUN060	M	33	LL	0	GT	AA	1.16	2.34	7.91
LEPFUN061	M	17	BT	0	TT	AG	1.16	2.00	0.82
LEPFUN062	M	62	BL	0	GG	AA	4.94	1.31	6.27
LEPFUN063	F	30	BL	0	GT	AG	0.67	0.85	0
LEPFUN064	M	31	LL	0	GG	AA	3.26	1.31	16.16
LEPFUN065	M	59	BL	0	GT	AA	0.94	0.62	1.17
LEPFUN066	M	38	BL	0	GT	GG	97.85	15.11	18.04
LEPFUN067	M	31	LL	0	GT	AA	3.04	1.08	5.46
LEPFUN068	M	60	BL	0	GT	AA	NA	4.06	NA
LEPFUN069	F	20	BT	0	GG	AA	3.43	4.06	11.17
LEPFUN070	F	51	BT	0	NA	NA	1.07	0.74	0
LEPFUN071	M	46	LL	0	NA	NA	1.65	2.23	6.98
LEPFUN072	M	58	BL	0	NA	NA	1.25	1.31	1.89
LEPFUN073	F	34	BT	0	NA	NA	1.87	1.08	1.08
LEPFUN074	F	35	BT	0	GT	AA	3.61	7.74	6.57
LEPFUN075	M	57	BT	0	NA	NA	1.87	1.20	0.82
LEPFUN076	F	18	BT	0	GG	AG	3.95	6.36	3.58
LEPFUN077	M	69	BT	0	GG	AG	6.73	10.28	5.96
LEPFUN078	M	60	BT	0	NA	NA	0.63	1.77	8.22
LEPFUN079	M	31	BL	0	GT	AG	1.43	0.97	2.82
LEPFUN080	M	34	LL	0	NA	NA	13.08	3.84	4.86
LEPFUN081	M	37	LL	0	NA	NA	5.84	1.77	19.27
LEPFUN082	F	59	BT	0	NA	NA	2.22	5.21	1.34
LEPFUN083	M	49	LL	0	NA	NA	6.47	1.08	9.68
LEPFUN084	F	26	LL	0	NA	NA	3.09	1.77	7.91

* Gender: F= Female; M= Male

** Reaction status: 1= reaction; 0= no reaction
